# Hybrid Forward-Backward Ray Tube Propagation Model for Deterministic Multipath Channel Prediction

**DOI:** 10.3390/s26185886

**Published:** 2026-09-17

**Authors:** Qi Yao, Zhongyu Liu, Lixin Guo

**Affiliations:** School of Physics, Xidian University, Xi’an 710071, China; one_seven_yq@163.com (Q.Y.); lxguo@xidian.edu.cn (L.G.)

**Keywords:** deterministic propagation prediction, forward–backward ray tube tracing, multipath propagation, reflection, refraction, diffraction, diffuse scattering, ray tracing, channel measurement

## Abstract

Deterministic propagation prediction faces a fundamental trade-off: the image method is exact but single-mechanism, while the shooting and bouncing ray method is flexible yet suffers from path omissions due to discrete sampling. This paper proposes the hybrid forward–backward ray tube propagation model (HFB-RTPM), which decouples the exhaustive forward construction of a ray tube tree from the backward path screening at the receiver, handling reflection, refraction, diffraction, and diffuse scattering within a unified framework. A quasi-3D extension and a dual-lobe diffuse scattering model are further introduced. Simulations show 99.8% reflection path completeness (RMSE 0.05 dB) and 100% diffraction completeness under all conditions. With diffuse scattering, the mmWave NLoS RMSE drops from 23.9/16.2 dB to 6.9/6.8 dB at 28/73 GHz, with a single model configuration valid across both frequency bands. In field measurements at 2.3–5.9 GHz across dense urban blocks, the simulation-to-measurement RMSE ranges from 5.6 to 8.5 dB. For large-scale coverage, HFB-RTPM is two orders of magnitude faster than the image method. The proposed method achieves accuracy, efficiency, and physical completeness for deterministic multipath prediction in 6G wireless networks.

## 1. Introduction

Sixth-generation (6G) wireless communication systems impose unprecedented accuracy requirements on channel modeling: radio wave propagation in the millimeter-wave and sub-millimeter-wave bands is highly sensitive to geometric details and material electromagnetic parameters, and statistically fitted path loss models can no longer meet the demand for space-time-frequency multi-dimensional channel information in fine-grained network planning and beam management. Deterministic propagation prediction methods have therefore become the industry consensus [1,2]. Multipath propagation in complex environments involves four fundamental electromagnetic interaction mechanisms, namely reflection, refraction, diffraction, and diffuse scattering, and their complete modeling constitutes the physical foundation for achieving high-accuracy deterministic prediction.

However, deterministic propagation prediction faces a core contradiction: the completeness of propagation mechanism modeling and computational feasibility cannot be simultaneously achieved in existing methods. The image method provides exact solutions for reflection paths on smooth surfaces through geometrical optics principles [3]. However, its applicability is limited to ideal reflective surfaces and cannot handle propagation mechanisms such as refraction, diffraction, and diffuse scattering. In the simulations of this paper, the image-method-based 2D prediction model (IM-2D), which computes the closed-form solutions of the reflection paths on smooth surfaces from geometrical optics, serves as the exact reference for path completeness and received-power accuracy in purely reflective scenarios, while its inability to treat refraction, diffraction, and diffuse scattering is precisely the limitation that motivates the unified multi-mechanism framework proposed in this work. The shooting and bouncing ray (SBR) method searches for propagation paths by densely launching rays from the transmitter, offering greater flexibility [4]. Yet its inherent discrete sampling nature leads to two fundamental problems: first, path omission and power errors caused by the reception sphere approximation [5]; second, computational overhead that grows exponentially with the number of rays and scene complexity [6]. Addressing the latter issue, researchers have proposed general acceleration techniques such as spatial partitioning structures and ray caching [7,8], as well as image-space-based preprocessing methods [9], which partially alleviate the computational bottleneck but do not fundamentally resolve the intrinsic contradiction between path completeness and computational efficiency [10,11].

In diffraction modeling, based on the geometrical theory of diffraction and the uniform theory of diffraction [12], various diffraction implementation schemes have been introduced into ray tracing frameworks [13]. However, existing methods typically rely on discretely launched rays for approximate diffraction path searching, and the inherent angular discretization errors lead to substantial omission of higher-order diffraction paths [14]. This problem is particularly pronounced in high-order reflection-diffraction coupling scenarios [15]. In refraction modeling, the computation of transmission rays based on Snell’s law is well established [16], but the complexity of path coupling in multi-layer dielectric penetration grows rapidly under high-order conditions, and traditional methods struggle to systematically manage multi-order refraction paths.

In diffuse scattering modeling, the scattering effects of rough surfaces are critical for non-line-of-sight (NLoS) coverage in the millimeter-wave band [17]. Existing studies have shown that diffuse scattering contributes significantly to received power in urban microcellular and indoor scenarios [18], and also affects channel delay spread and angular spread [19]. However, existing deterministic methods mostly treat scattering through independent superposition, lacking a unified parameterization framework that integrates reflection, diffraction, and other mechanisms [20]. Large-scale measurements in the millimeter-wave band further indicate [21,22] that the energy diffusion phenomenon under NLoS propagation conditions imposes higher requirements on the completeness of deterministic models.

Ray tracing has been widely applied to channel characterization and propagation prediction in indoor [23], urban [19], vehicular communication [24] scenarios, as well as special environments such as high-speed railways and tunnels [25]. Methods such as deep learning assistance [26] and reconfigurable intelligent surface modeling [27] have further expanded its application boundaries. In addition, quasi-three-dimensional propagation modeling based on the cylindrical symmetry assumption [28] significantly reduces computational complexity by decoupling the horizontal plane and the vertical dimension [29]. However, most existing quasi-3D methods support only the reflection mechanism and have not formed a unified modeling framework with refraction, diffraction, and diffuse scattering. These methods are mostly oriented toward specific scenarios or specific propagation mechanisms, and have not yet established a unified deterministic propagation prediction framework that can simultaneously support all four mechanisms of reflection, refraction, diffraction, and diffuse scattering [30].

To address the above challenges, this paper proposes the hybrid forward–backward ray tube propagation model, termed HFB-RTPM, a complete deterministic propagation prediction framework that integrates environment modeling, hybrid forward–backward ray tube tracing, quasi-three-dimensional path extension, and field computation into a unified pipeline. The main contributions are summarized as follows:

First, a hybrid forward–backward framework is proposed, which decouples the exhaustive construction of ray tube trees (forward) from the receiver-end backtracking and filtering of paths (backward), thereby fundamentally eliminating path omissions caused by discrete sampling and guaranteeing path completeness.

Second, within a unified ray tube generation and counter management framework, the systematic coupling of four mechanisms, i.e., reflection, refraction, diffraction, and diffuse scattering, is realized, overcoming the limitation of traditional methods that can only handle single or partial mechanisms.

Third, a dual-lobe diffuse scattering modeling strategy is proposed, introducing statistical modeling of rough surface scattering within the deterministic framework, which significantly improves the prediction accuracy in millimeter-wave NLoS scenarios.

Fourth, a low-complexity method for extending two-dimensional ray paths to quasi-three-dimensional scenarios is proposed, recovering vertical-dimensional propagation information while maintaining computational efficiency.

Fifth, through mechanism-by-mechanism simulations and four-band measurement validation, the comprehensive advantages of HFB-RTPM in the three dimensions of accuracy, efficiency, and physical completeness are thoroughly verified.

## 2. Electromagnetic Modeling of the Propagation Environment

High-accuracy deterministic propagation prediction relies on the precise electromagnetic characterization of the propagation environment. This section establishes the foundation for two-dimensional deterministic propagation modeling from two aspects: the electromagnetic parameters of building materials and the geometric characterization of the environment.

### 2.1. Electromagnetic Parameters of Building Materials

In ray tracing-based propagation prediction, the electromagnetic properties of the media in the environment, namely the relative permittivity $\varepsilon_{r}$ and conductivity σ, are key input parameters for calculating reflection, refraction, and energy attenuation of radio waves at various interfaces. This paper adopts the fitting model of electromagnetic parameters for building materials provided by ITU-R Recommendation P.2040-3 [31], which expresses the relative permittivity and conductivity as power-law functions of frequency:

(1) $$\begin{array}{cc} \varepsilon_{r} & =af^{b},\\ \sigma & =cf^{d}. \end{array}$$

where *f* is the frequency (in GHz), and *a*, *b*, *c*, *d* are the fitting coefficients corresponding to each material, with specific values listed in Table 1. This model is derived from curve fitting of extensive published measurements and mainly covers the electromagnetic properties of typical building materials from 1 MHz to 100 GHz. Recent research [32] shows that when this model is extended to the 260 GHz band, the permittivity can still be modeled as a frequency-independent constant, while the frequency dependence of conductivity follows the same $cf^{d}$ form, validating the applicability of the ITU model in the sub-millimeter-wave band.

By substituting the fitting coefficients from Table 1 into Equation (1), the electromagnetic parameters of each material at any given frequency can be obtained. Taking 3 GHz as an example, the relative permittivity and conductivity of typical building materials are shown in the last two columns of Table 1. These parameters will be used in the computation of complex reflection coefficients and complex transmission coefficients for various propagation mechanisms in subsequent simulations.

Table 1 shows that the *b* values of all materials are zero (relative permittivity is frequency-independent), whereas *d* is significantly non-zero (conductivity varies markedly with frequency), which must be considered in high-frequency band propagation prediction.

### 2.2. Geometric Characterization of the Two-Dimensional Propagation Environment

In two-dimensional deterministic propagation modeling, the environment is abstracted as a collection of dielectric interfaces. The boundary of each object is represented as a directed line segment, referred to as a face, with the set denoted as $F=\{f_{1},f_{2},\ldots ,f_{N}\}$. Each face $f_{i}$ is defined in terms of its spatial position and orientation by the coordinates of its two endpoints $p_{i,1},p_{i,2}\in R^{2}$ and the unit normal vector ${\hat{n}}_{i}$, and carries the material types $M_{i}^{+}$ and $M_{i}^{-}$ on both sides of the normal direction, as well as the surface roughness parameter $\rho_{i}$ (used to trigger the diffuse scattering mechanism). The directionality of the normal vector ensures the correctness of determining the incident, reflected, and transmitted directions in the Fresnel equations, while the material properties on both sides directly determine the computation of reflection and transmission coefficients.

The common endpoint of two intersecting faces is referred to as a corner, with the set denoted as $C=\{c_{1},c_{2},\ldots ,c_{K}\}$. Each corner $c_{j}$ is associated with the two intersecting faces $f_{j,1}$ and $f_{j,2}$, providing the wedge geometry parameters for the computation of diffraction coefficients in the uniform theory of diffraction and enabling fast retrieval of diffraction sources at runtime.

In this representation, an arbitrary boundary is discretized into directed straight-line face segments, so a curved obstacle is approximated by a polygonal chain of faces, whose approximation error decreases as the face size is reduced. When the radius of curvature of an obstacle becomes comparable to the wavelength (on the order of several centimeters), the straight-line facet approximation introduces a geometric error, which can be reduced by refining the facet discretization or by adopting a local higher-order boundary approximation; this is a discretization-accuracy issue rather than a limitation of the path-completeness property of the tracing algorithm.

Based on the above representation, the two-dimensional propagation environment is defined as the combination of the geometric sets F and C together with the electromagnetic parameter set $P=\{\varepsilon_{r}\left(f\right),\sigma \left(f\right)\}$. This representation uniformly maps the geometric structure and electromagnetic properties of the real-world scenario onto an abstract model that can be processed by the ray tracing algorithm, thereby providing the foundation for the accurate computation of multi-mechanism propagation paths.

**Figure 1:**
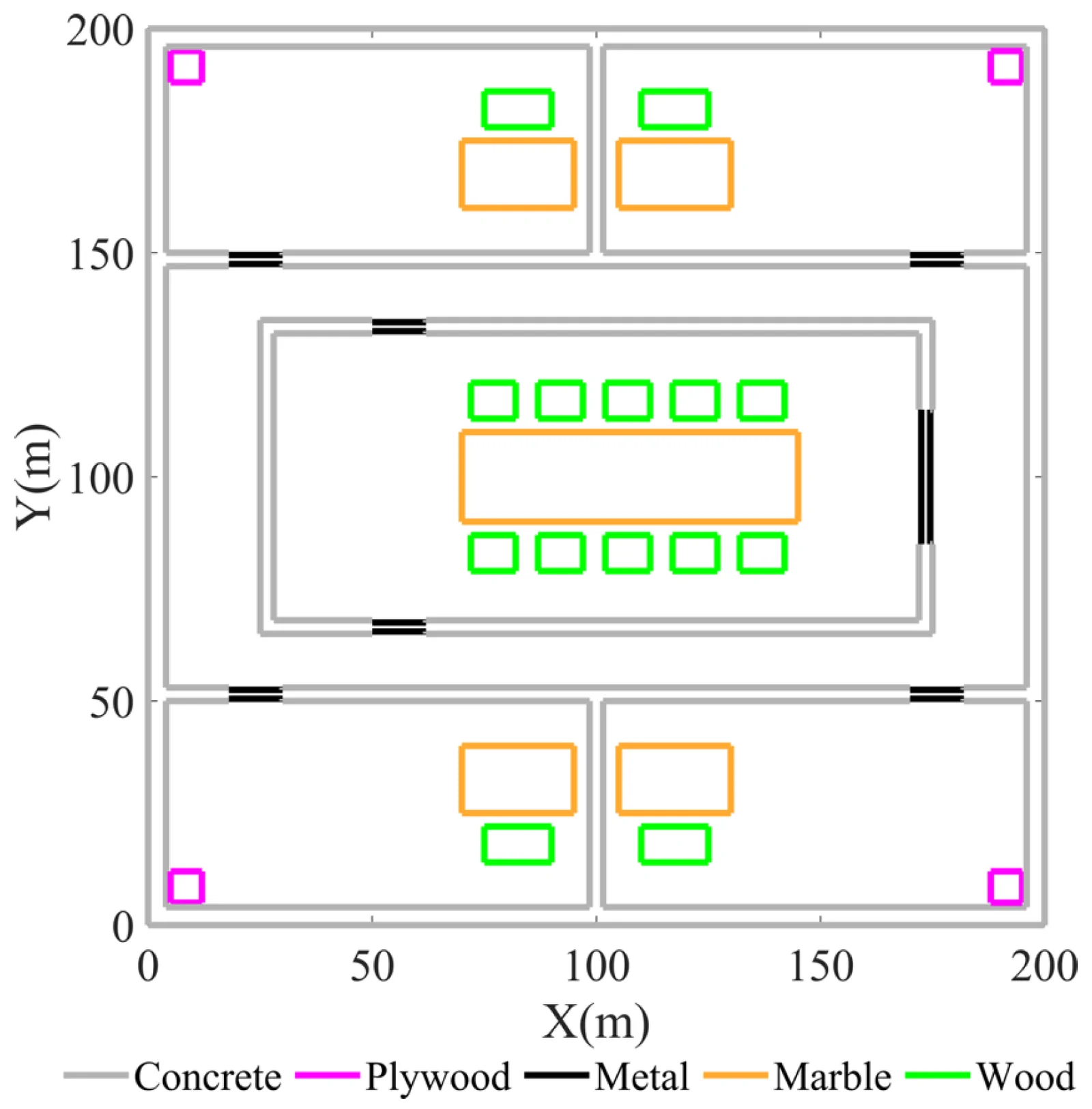
Geometric modeling result of a typical two-dimensional indoor scenario.

Figure 1 shows a typical indoor scenario modeled using this approach: the scenario is a 200 m × 200 m two-dimensional plane, containing 180 geometric elements and 5 ITU materials (concrete, wood, plywood, marble, metal). The physical boundaries in the scenario, such as walls and floors, are discretized into the set of directed faces F, each carrying a normal vector and material properties on both sides; the intersections of adjacent faces form the set of corners C; and each face is assigned the corresponding frequency-dependent electromagnetic parameters P according to its material type.

## 3. HFB-RTPM Propagation Prediction Framework

Based on the two-dimensional environmental electromagnetic modeling described in the previous section, this section presents the HFB-RTPM propagation prediction framework, whose core is the hybrid forward–backward ray tube tracing (HFB-RTT) algorithm. The framework integrates three key components: first, the HFB-RTT algorithm (Section 3.1, Section 3.2 and Section 3.6), which decouples the exhaustive construction of ray tube trees from the on-demand backtracking at the receiver, eliminating path omissions caused by discrete sampling at the architectural level; second, unified multi-mechanism ray tube generation rules (Section 3.3, Section 3.4 and Section 3.5) that systematically handle four propagation interactions, namely reflection, refraction, diffraction, and diffuse scattering, within the HFB-RTT algorithm; and third, quasi-three-dimensional path extension and electric field computation (Section 3.7 and Section 3.8) that recover vertical-dimension propagation information while maintaining computational efficiency and complete the mapping from path geometry to received power.

### 3.1. Design Principle and Overall Flow of the Hybrid Framework

The core idea of HFB-RTT is the separated design of forward recursive encoding and backward on-demand reconstruction. In the forward phase, the algorithm uses the transmitting antenna as the root node and organizes all possible propagation interactions in the environment into a ray tube tree through recursive collision detection and ray tube splitting. In the backward phase, the algorithm starts from the receiving antenna and traces upward level by level along the parent pointers of the ray tube tree, restoring the complete geometric and electromagnetic information only for paths that actually reach the receiver, thereby avoiding the redundant computation that characterizes traditional unidirectional methods, where large numbers of rays are launched in directions with no receivers.

**Figure 2:**
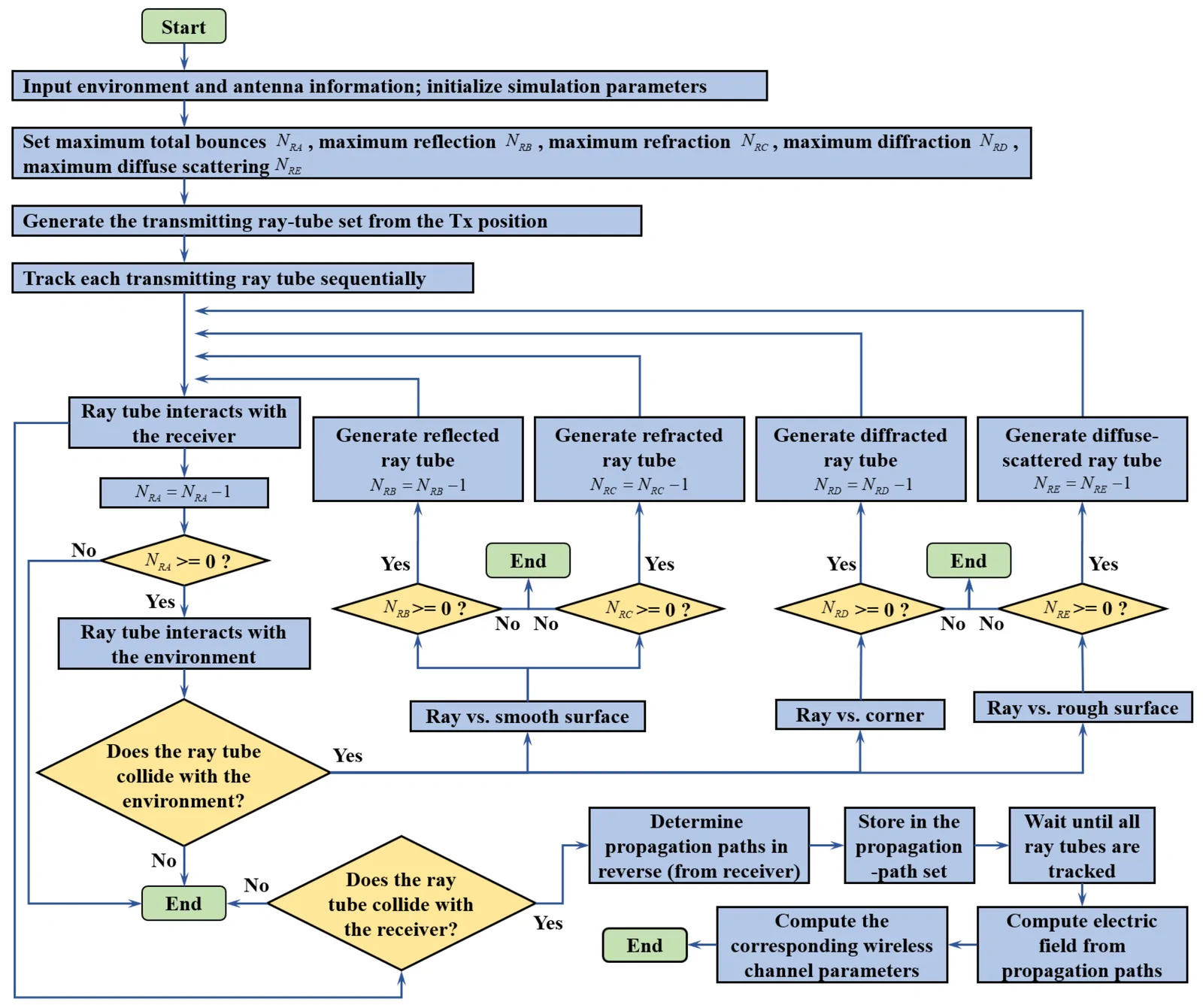
Overall flowchart of the HFB-RTPM framework. (Green rounded boxes denote the start and end states of the process, blue rectangles denote the processing steps and the generation of new ray tubes, and yellow diamonds denote the conditional judgments.).

Figure 2 presents the overall solution flow of HFB-RTPM, where green rounded boxes denote the start and end states of the process, blue rectangles denote the processing steps and the generation of new ray tubes, and yellow diamonds denote the conditional judgments. The flow consists of the following eight sequential steps.

**Step 1: Initialization.** Load the geometric and electromagnetic information of the two-dimensional scenario, set the positions of the transmitting and receiving antennas, and configure five simulation control parameters, including the maximum number of bounces $N_{RA}$ and the maximum allowed numbers of reflection, refraction, diffraction, and diffuse scattering events $N_{RB}$, $N_{RC}$, $N_{RD}$, and $N_{RE}$. Generate the initial set of ray tubes according to the transmitting antenna radiation pattern and place them into the pending queue.

**Step 2: Loop scheduling.** Dequeue a ray tube from the pending queue and enter the processing flow; if the queue is empty, all ray tubes have been traced, and the flow proceeds to Step 8.

**Step 3: Receiver interaction and bounce count update.** Determine whether the fan-shaped region of the ray tube covers the receiver position; if so, record the path. Then decrement the remaining bounce count $N_{RA}$ by 1; if $N_{RA}$ reaches zero, terminate the current processing and return to Step 2.

**Step 4: Collision detection.** Compute the collision results of the ray tube with all faces and corners in the scene. If no valid collision exists, proceed to Step 5; otherwise, proceed to Step 6.

**Step 5: Escape handling.** When the ray tube does not collide with any face or corner, its propagation direction may still cover the receiver position; therefore, the potential path is checked and recorded again. The ray tube is then terminated, and the flow returns to Step 2.

**Step 6: Generation of new ray tubes.** Based on the geometric type and material properties of the collided object, child ray tubes are generated along four parallel branches: a smooth line segment triggers reflection and decrements $N_{RB}$ by 1, a dielectric interface triggers refraction and decrements $N_{RC}$ by 1, a corner triggers diffraction and decrements $N_{RD}$ by 1, and a rough line segment triggers diffuse scattering and decrements $N_{RE}$ by 1. Each branch generates ray tubes and enqueues them only when the corresponding remaining count is greater than zero. The new ray tubes inherit the updated counters from the parent ray tube and record parent pointers to maintain the connectivity of the tree structure. The construction rules for each mechanism are detailed in Section 3.3, Section 3.4 and Section 3.5.

**Step 7: Iterative advancement.** Return to Step 2, dequeue the next ray tube from the queue, and continue processing until the queue is empty.

**Step 8: Backward path reconstruction and channel parameter computation.** After the pending queue is emptied, all recorded paths are traced backward along the parent pointers to recover the complete propagation sequence and the geometric coordinates of each interaction point. Based on the geometric path length, propagation mechanism type, and material electromagnetic parameters, the complex electric field and received power of each path are computed and superposed to obtain the total received power at the receiver. Finally, the required wireless channel parameters are output. The detailed procedures of backward reconstruction and electric field computation are presented in Section 3.6 and Section 3.8, respectively.

In the above procedure, $N_{RA}$ constrains the total propagation order of a single path, and $N_{RB}$, $N_{RC}$, $N_{RD}$, and $N_{RE}$ respectively constrain the recursion depth of each physical mechanism, effectively controlling the size of the state space while ensuring model completeness.

The collision detection stage involves intersection tests between ray tubes and all faces and corners, representing the most computationally intensive part of the flow. To address this, the present work introduces spatial acceleration structures from computer graphics, adopting a bounding volume hierarchy (BVH) based on the surface area heuristic (SAH) to manage the sets of faces and corners in the scene. This reduces the average complexity of a single intersection test from linear to logarithmic order, providing efficiency guarantees for recursive collision detection in large-scale scenarios.

### 3.2. Construction of Transmitting Ray Tubes

**Figure 3:**
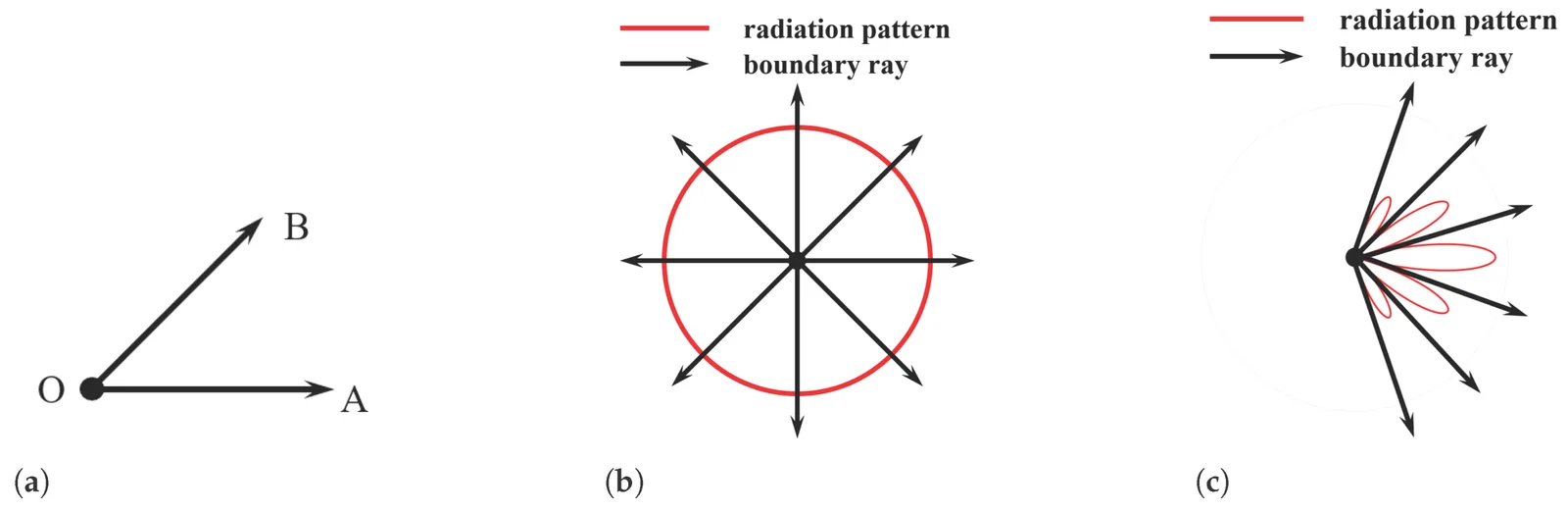
Geometry of 2D transmitting ray tubes and antenna radiation discretization: (**a**) a single transmitting ray tube; (**b**) ray tube bundle for an omnidirectional antenna (uniform 360° partition); (**c**) ray tube bundle for a directional antenna (within the main-lobe window).

The ray tube tree takes the transmitting ray tube as its root node; constructing these initial ray tubes is the starting point of the entire hybrid framework. In the two-dimensional plane, a ray tube is defined as a fan-shaped region bounded by two boundary rays, with its vertex located at the transmitting antenna, as shown in Figure 3a. To facilitate a unified description of the geometric construction of subsequent propagation mechanisms, the transmitting ray tube in Figure 3a is denoted as *O*–$\vec{OA}$–$\vec{OB}$, where the vertex *O* is the transmitting antenna position $T_{A}$, and *A* and *B* are reference points on the two boundary rays. Let the transmitting antenna position be $T_{A}$ and the angular discretization interval be Δθ. The fan-shaped region covered by the *i*-th transmitting ray tube can then be formally expressed as

(2) $$R_{i}=\left\{r\in R^{2}:\left|∠\left(r-T_{A}\right)-\theta_{i}\right|\leq \frac{\Delta \theta }{2}\right\},\;\theta_{i}=i\Delta \theta$$

where r is an arbitrary position vector in the two-dimensional plane, ∠(·) denotes the azimuth angle of the vector relative to the reference direction, and $\theta_{i}$ is the central direction angle of the *i*-th ray tube. The angular span of the fan-shaped region is determined by the horizontal-plane radiation pattern of the antenna. For an omnidirectional antenna, fan-shaped ray tubes must be uniformly generated over the full 360° range to form complete annular coverage; for a directional antenna, energy is concentrated in the main-lobe direction, and ray tube bundles are generated only within the limited angular window covered by the main lobe. This main-lobe-only generation is the default configuration and rests on the approximation that the main-lobe energy dominates the total radiated power. For a practical directional antenna with non-negligible side lobes, the side-lobe energy can be accounted for by treating the side lobes as additional off-boresight transmitting sources, in which case Φ is extended to the union of the main-lobe window and the significant side-lobe windows. Because the forward ray-tube-tree construction and the backward reconstruction are intrinsically independent of the angular distribution of the transmitting sources, this extension requires no change to the algorithm itself. Let the total angular span of the radiation coverage be Φ; for an omnidirectional antenna Φ=2π, and for a directional antenna $\Phi =\theta_{\mathrm{HPBW}}$, where $\theta_{\mathrm{HPBW}}$ is the half-power beamwidth of the main lobe. The number of initial transmitting ray tubes is then

(3) $$N_{0}=\left\lceil \Phi /\Delta \theta \right\rceil$$

The angular discretization interval Δθ does not directly affect the path prediction accuracy, since HFB-RTT can recover all propagation paths admitted by the discretized environment model without path omission caused by angular sampling, through ray tube splitting and backward path reconstruction. However, Δθ directly determines the computational cost, and both excessively large and excessively small values lead to increased computation: when Δθ is too large, the fan-shaped coverage of a single ray tube is wide, and one collision detection may simultaneously span multiple interaction objects, forcing the algorithm to split it into several sub-ray tubes for separate processing, with the proliferation of sub-ray tubes actually aggravating the computational burden; when Δθ is too small, the number of initial ray tubes $N_{0}$ determined by Equation (3) increases accordingly, and the total number of collision detections rises correspondingly. Therefore, Δθ has a favorable value range that balances computational efficiency; based on numerical experiments, this paper adopts $\Delta \theta =22.5^{\circ }$.

The ray-tube splitting triggered by a large Δθ is performed as follows. When a single fan-shaped ray tube intersects several interaction objects in one collision detection, the algorithm partitions the fan-shaped angular domain into a set of mutually non-overlapping sub-fans, using the boundary directions of the interaction objects as the partition boundaries, so that each sub-fan corresponds to exactly one interaction object. Concretely, the angular interval $\left[\theta_{i}-\Delta \theta /2,\;\theta_{i}+\Delta \theta /2\right]$ is subdivided in a monotonic manner at the projected boundary directions of the collided objects; contiguous angular intervals that fall on the same face segment are merged into a single sub-ray tube, whereas intervals that span multiple corners are further subdivided at the wedge boundaries of those corners. Each sub-ray tube inherits the counters and the parent pointer of the parent ray tube and is then processed recursively. This tree-structured splitting guarantees that distinct interaction objects lying within one wide fan are not lost under a large Δθ, and it is the mechanism by which HFB-RTT replaces the dense discrete launching of SBR with an architectural guarantee of path completeness.

Each initial transmitting ray tube is assigned the full set of simulation control parameters ($N_{RA}$, $N_{RB}$, $N_{RC}$, $N_{RD}$, $N_{RE}$) and then placed into the pending queue. The fan-shaped regions covered by the transmitting ray tubes are mutually non-overlapping and independent, and their subsequent collision detection and ray tube splitting processes have no data dependencies, thus allowing parallel processing on multi-core architectures. As the root nodes of the ray tube tree, transmitting ray tubes carry no parent pointers; all subsequent ray tubes at deeper levels record parent pointers at the time of generation, providing the topological basis for backward path reconstruction, as detailed in Section 3.6.

### 3.3. Construction of Reflection and Refraction Ray Tubes

When radio waves propagate in the environment, the interaction with dielectric interfaces is the most fundamental propagation mechanism. When a ray tube impinges on a dielectric interface, reflected and transmitted waves are generated simultaneously, sharing the same collision event and incident ray tube, with only the propagation direction and complex amplitude determined according to their respective laws. The geometric construction of reflection and refraction is based on the method of images and Snell’s law, respectively.

**Figure 4:**
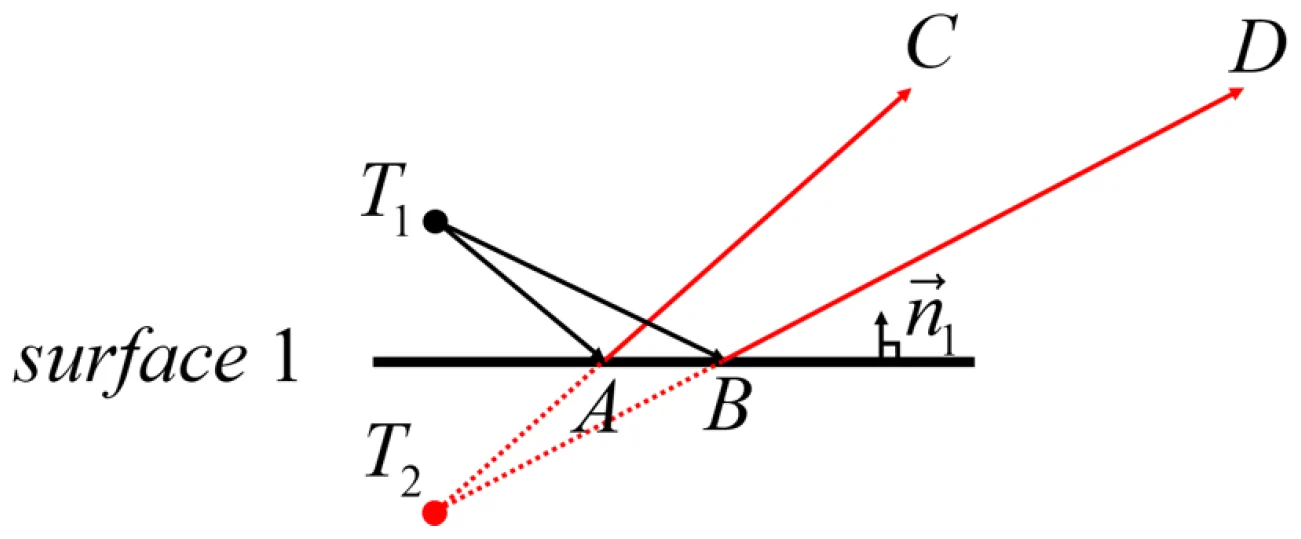
Illustration of reflected ray tube construction. (The red solid arrows denote the reflected boundary rays, the red dotted lines their backward extensions to the image point $T_{2}$, the black lines the incident rays, the thick black line the reflecting surface, and the short black arrow its unit normal).

When an effective collision occurs between a ray tube and a smooth line segment (face) in the scene, the algorithm triggers the construction of a reflected ray tube. According to the principles of geometrical optics, after the incident ray tube $T_{1}$–$\vec{T_{1}A}$–$\vec{T_{1}B}$ effectively collides with the smooth face surface1, the reflected directions of the two boundary rays are determined according to the law of reflection. The backward extensions of the reflected rays intersect at the image point $T_{2}$ of the incident ray tube with respect to the reflecting interface, thereby determining the vertex position and boundary directions of the reflected ray tube, producing the reflected ray tube $T_{2}$–$\vec{AC}$–$\vec{BD}$, whose geometric construction is illustrated in Figure 4.

The magnitude of the reflected field is determined by the complex reflection coefficient of the reflecting interface, which is given by the Fresnel equations and depends on the incident angle and the complex relative permittivities of the media on both sides of the interface; the complex relative permittivity is jointly determined by the relative permittivity, conductivity, and frequency as described in Section 2.1. The reflected ray tube inherits the propagation attributes of the parent ray tube, with its complex amplitude corrected by the complex reflection coefficient and the remaining reflection count $N_{RB}$ decremented by 1.

**Figure 5:**
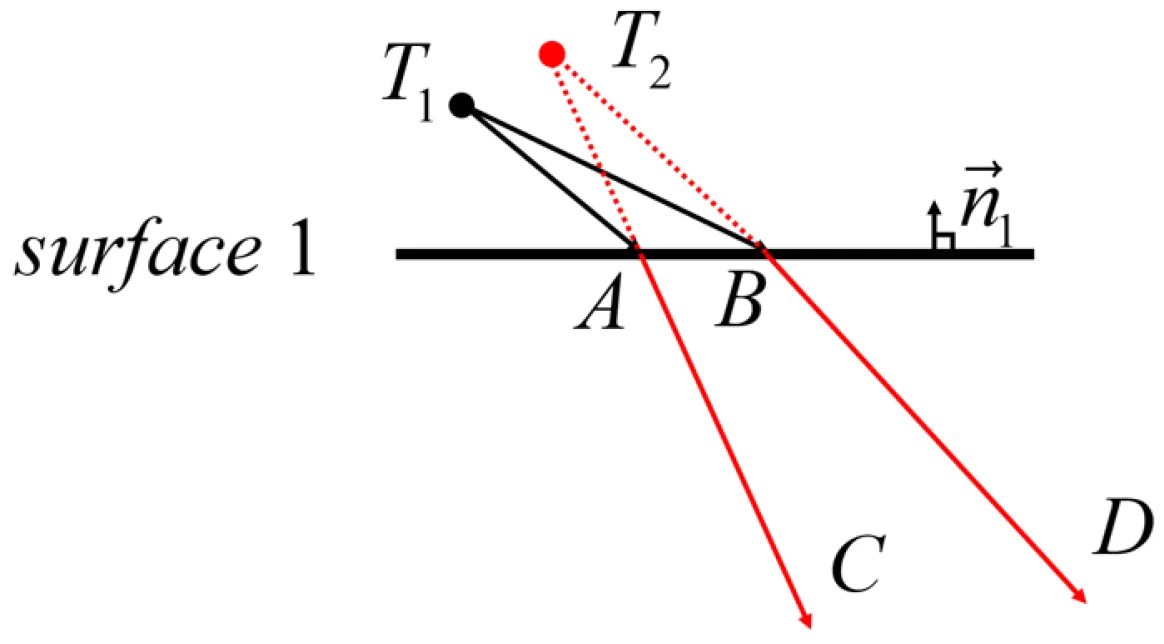
Illustration of refracted ray tube construction. (The red solid arrows denote the refracted boundary rays, the red dotted lines their backward extensions to the point $T_{2}$, the black lines the incident rays, the thick black line the dielectric interface, and the short black arrow its unit normal).

When the collided face is a boundary between two media, the algorithm constructs a refracted ray tube according to Snell’s law simultaneously with the generation of the reflected ray tube. The construction procedure for the refracted ray tube is as follows: after the incident ray tube $T_{1}$–$\vec{T_{1}A}$–$\vec{T_{1}B}$ effectively collides with the dielectric interface surface1, a refracted ray tube $T_{2}$–$\vec{AC}$–$\vec{BD}$ is produced. The refraction angle is jointly determined by the incident angle and the electromagnetic parameters of the media on both sides. The intersection point $T_{2}$ of the backward extensions of the refracted rays lies on the same side of the interface as $T_{1}$, and its geometric construction is illustrated in Figure 5. Specifically, when radio waves propagate from a medium with higher permittivity to one with lower permittivity and the incident angle exceeds the critical angle, the transmitted wave vanishes and total internal reflection occurs at the interface; in this case, no refracted ray tube is generated.

The refracted ray tube inherits the propagation attributes of the parent ray tube. Its complex transmission coefficient is given by the Fresnel transmission formulas, depends on the complex relative permittivities of the media on both sides and the incident angle, and satisfies the energy conservation relationship with the complex reflection coefficient. The propagation direction of the refracted ray tube is corrected according to Snell’s law, and the remaining refraction count $N_{RC}$ is decremented by 1 to constrain the upper bound of the refraction path order. Penetration through multi-layer dielectrics causes the energy to attenuate progressively along the transmission chain.

### 3.4. Construction of Diffraction Ray Tubes

**Figure 6:**
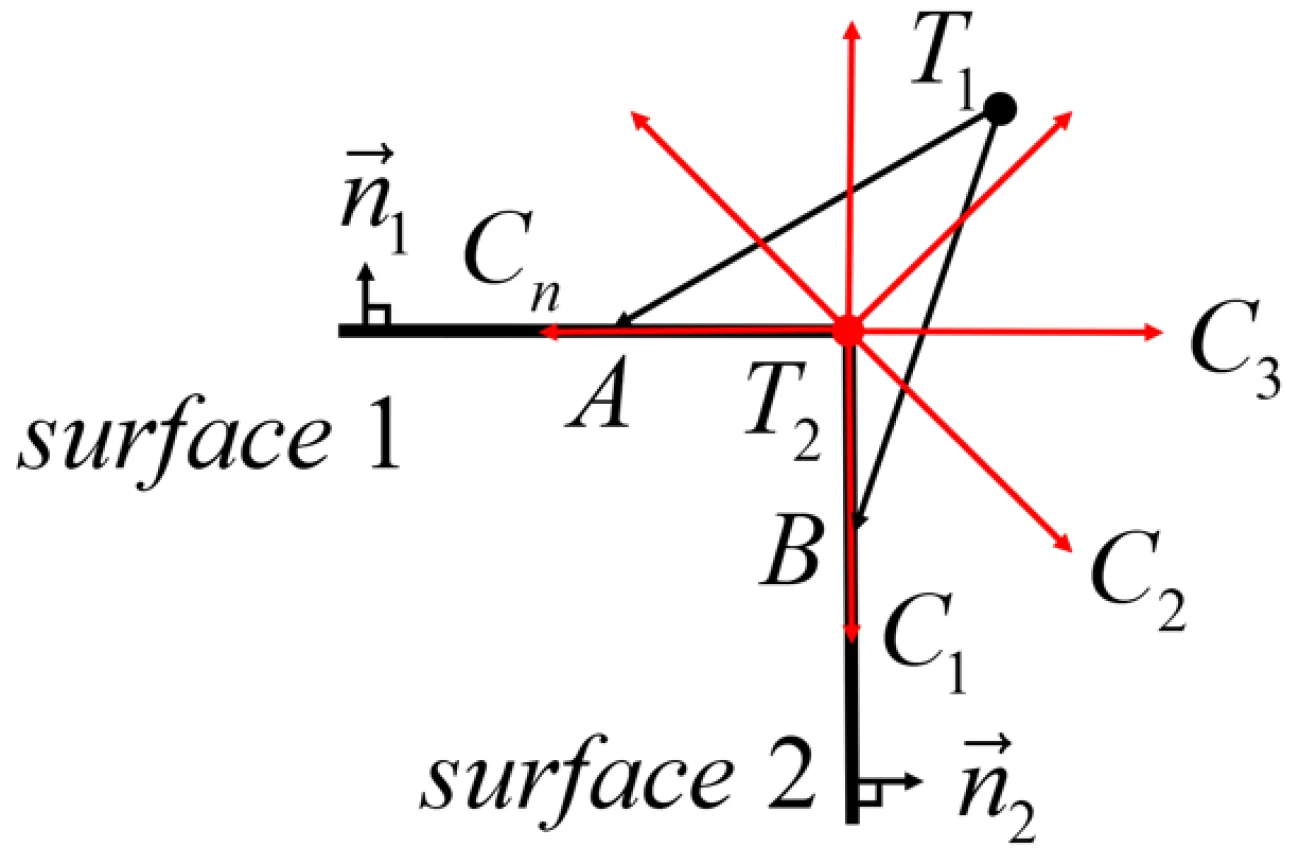
Illustration of diffracted ray tube construction. (The red arrows denote the diffracted rays in different angular directions, the red star the diffraction corner $T_{2}$, the black lines the incident rays, the thick black lines the two faces forming the wedge, and the short black arrows the unit normals of the two faces).

When the propagation path is obstructed by building edges, diffraction becomes a key source of NLoS signals, and its processing relies on the corner information in the scene. When a ray tube impinges on a corner $T_{2}$ formed by two faces, the algorithm generates a set of diffracted ray tubes with an angular interval (taken as 45° in this paper) within the fan-shaped region spanned by the corner, as shown in Figure 6, where each diffracted ray tube represents a discrete diffraction direction. Specifically, this set of diffracted ray tubes is denoted as $T_{2}$–$\vec{T_{2}C_{1}}$–$\vec{T_{2}C_{2}}$, $T_{2}$–$\vec{T_{2}C_{2}}$–$\vec{T_{2}C_{3}}$, …, $T_{2}$–$\vec{T_{2}C_{n-1}}$–$\vec{T_{2}C_{n}}$. All corners in the scene that may give rise to diffraction have been identified and stored in the corner database during the initialization phase, and a pre-built edge topology lookup table is constructed so that the runtime generation of diffracted ray tubes can be accomplished with constant time complexity.

The magnitude of the diffracted field is described by the uniform theory of diffraction (UTD). The diffraction coefficient is jointly determined by the angle between the incident direction and one side face of the edge, the angle between the incident direction and the edge, and the wedge angle, and includes terms such as the reflection coefficients of the faces on both sides of the edge, transition functions, and distance parameters. In this paper, the wedge angle is determined by the two line segments that form the corner and their normal vectors. Each diffracted ray tube inherits the propagation attributes of the parent ray tube, with its complex amplitude corrected by the corresponding UTD diffraction coefficient and $N_{RD}$ decremented by 1.

### 3.5. Construction of Diffuse Scattering Ray Tubes

The aforementioned reflection, refraction, and diffraction mechanisms are all established on the assumption of smooth interfaces; however, the roughness inherent in real surfaces induces diffuse scattering, which has a significant impact on NLoS coverage at high frequencies. When a ray tube impinges on a face, HFB-RTT employs the Rayleigh roughness criterion to determine whether it is a rough surface: let Δh be the standard deviation of the surface height fluctuation; when $\Delta h>\lambda /\left(8\sin\theta_{i}\right)$ is satisfied, the face is classified as a rough surface, where λ is the wavelength and $\theta_{i}$ is the incident angle. For collisions on rough surfaces, the algorithm divides the collision line segment into multiple equally spaced diffuse scattering centers according to the specified maximum diffuse scattering line segment length $l_{ds}$. The position of the *i*-th diffuse scattering center is

(4) $$S_{i,\mathrm{center}}=P_{0}+\left(i-0.5\right)l_{s}\hat{v},\;i=1,2,\ldots ,m$$

where $P_{0}$ is the starting point of the collision line segment, $\hat{v}$ is the unit vector from the start to the end of the segment, *L* is the length of the collision line segment, $m=\lfloor L/l_{ds}\rfloor +1$ is the number of diffuse scattering centers, and $l_{s}=L/m$ is the actual discrete length. Each diffuse scattering center generates a set of diffuse scattering ray tubes at discrete angles within its scattering range.

**Figure 7:**
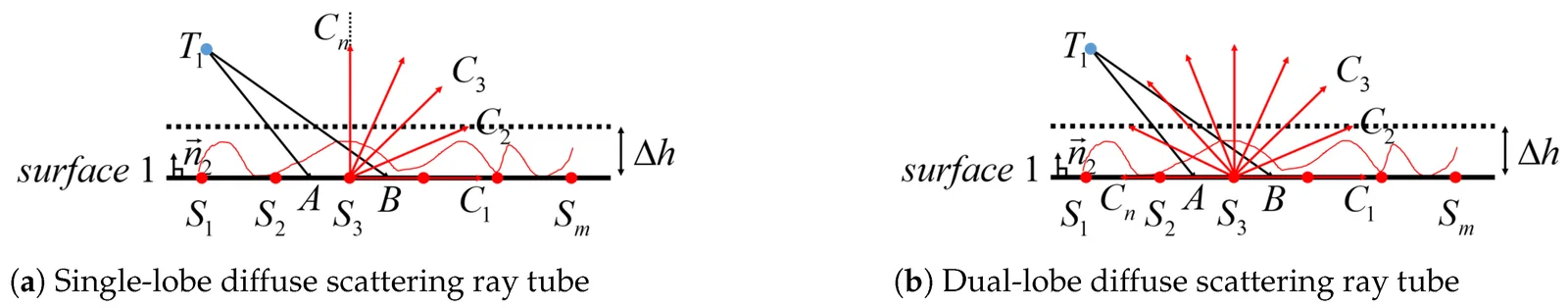
Schematic of single-lobe and dual-lobe diffuse scattering ray tubes. (The blue dot denotes the transmitter, the red dots the diffuse scattering centres on the rough surface, the red curves the surface height fluctuation, the black dashed line the reference plane with height deviation Δh, the red arrows the generated scattering ray tubes, and the black lines the incident rays).

This paper supports two diffuse scattering lobe models: single-lobe diffuse scattering takes the specular reflection direction as the center and extends over a specified angular range on both sides; dual-lobe diffuse scattering forms one scattering lobe each in the specular reflection direction and the incident direction, corresponding respectively to the forward and backward scattering characteristics of rough surfaces, whose geometric constructions are illustrated in Figure 7. All diffuse scattering ray tubes inherit the propagation attributes of the parent ray tube, with their complex amplitudes corrected according to the scattering direction and $N_{RE}$ decremented by 1.

The diffuse scattering mechanism introduces the capability of statistical modeling of rough-surface scattering within the deterministic ray tube framework. By uniformly controlling the distribution of scattering energy between specular reflection and diffuse scattering through the diffuse scattering coefficient $S_{ds}$, this method can accurately characterize the continuous transition from smooth to rough surfaces within a single framework, thereby enhancing the modeling capability for high-frequency propagation characteristics of real physical environments.

### 3.6. Backward Path Reconstruction Based on the Ray Tube Tree

The forward tracing described above encodes all possible propagation interactions in the environment as a ray tube tree, but the complete propagation sequence of each multipath must be recovered on demand. After the forward tracing is completed, all ray tubes that can reach the receiver constitute the leaf nodes of the ray tube tree. Backward path reconstruction starts from these leaf nodes, traces upward level by level along the parent pointers to the root node, and reads the propagation mechanism type and geometric parameters recorded at each node during the backtracking process, thereby recovering the complete radio wave propagation sequence.

**Figure 8:**
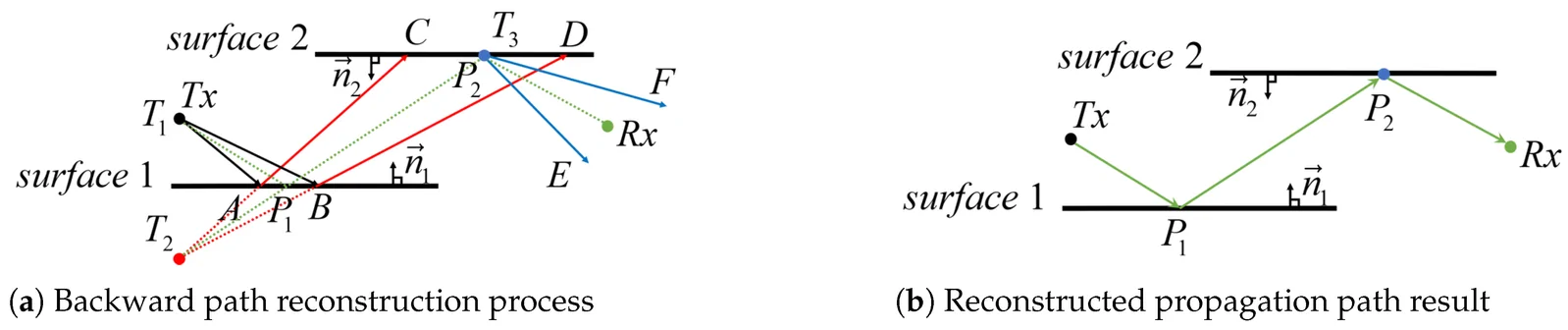
Schematic of backward path reconstruction based on the ray tube tree. (The black dot denotes the transmitter, the blue dot the diffuse scattering vertex, the green dot the receiver, the red solid arrows the reflected boundary rays, the red dotted lines their backward extensions to the image point, the green dotted arrows the backward reconstruction from the receiver, and the blue arrows the ray tubes generated at the scattering vertex).

As shown in Figure 8a, backward reconstruction starts from the receiver, successively connecting the diffuse scattering ray tube vertex and the reflection ray tube vertex until reaching the transmitter; Figure 8b shows the resulting reconstructed propagation path, whose propagation sequence is $\mathrm{Tx}\to P_{1}$ (reflection) $\to P_{2}$ (diffuse scattering) →Rx, where the transmitter (Tx) and the receiver (Rx) are the start and end points of the path, respectively.

Depending on the propagation mechanism, path reconstruction exhibits two geometric features: for transmitting ray tubes and ray tubes generated by diffraction and diffuse scattering (point-source type), the nodes of the propagation path are directly located at the vertex positions of the ray tubes; for ray tubes generated by reflection and refraction (interface type), the nodes of the propagation path lie on the interface line segment that generated the ray tube and must be solved through exact ray-segment intersection tests. This categorized treatment ensures the geometric accuracy of each path and avoids the path distortion introduced by the reception sphere approximation in conventional ray launching methods.

### 3.7. Quasi-Three-Dimensional Extension of Two-Dimensional Propagation Paths

The above modeling is performed entirely in a two-dimensional plane, whereas the actual wireless communication environment is inherently three-dimensional. To apply the two-dimensional prediction results to nearly realistic three-dimensional scenarios while maintaining computational efficiency, this paper proposes a path extension method based on the vertical independence assumption, which is suitable for propagation scenarios with pronounced cylindrical symmetry characteristics, such as urban canyons, indoor corridors, and densely built-up urban blocks. This method rests on three assumptions: all obstacles in the scenario can be idealized as cylinders extending infinitely in the vertical direction; the three-dimensional positions of the transmitting and receiving antennas are known, denoted as $\mathrm{Tx}=(x_{t},y_{t},z_{t})$ and $\mathrm{Rx}=(x_{r},y_{r},z_{r})$, respectively; and the propagation path in the horizontal plane and the propagation characteristics in the vertical plane can be approximately decoupled. Under these assumptions, a complete three-dimensional propagation path is decomposed into two orthogonal components: a horizontal projection path and a vertical connection path. Let the sequence of horizontal path interaction points obtained from two-dimensional tracing be

(5) $$\psi_{2D}=\left\{V_{0},V_{1},\ldots ,V_{n}\right\}$$

where $V_{0}=\left(x_{t},y_{t}\right)$ is the transmitting point, $V_{n}=\left(x_{r},y_{r}\right)$ is the receiving point, and the intermediate points $V_{i}$ are the locations where the path interacts with the environment through mechanisms such as reflection and diffraction. The three-dimensional path vertex sequence $\psi_{3D}=\left\{P_{0},P_{1},\ldots ,P_{n}\right\}$ satisfies $P_{i,x}=V_{i,x}$ and $P_{i,y}=V_{i,y}$, and its vertical coordinates are obtained through linear interpolation of the transmitting and receiving antenna heights:

(6) $$P_{i,z}=P_{0,z}+\frac{l_{i,2D}\left(P_{n,z}-P_{0,z}\right)}{l_{n,2D}},\;i=1,2,\ldots ,n-1$$

(7) $$l_{i,2D}=\sum_{j=1}^{i}\left|V_{j}-V_{j-1}\right|,\;i=1,2,\ldots ,n$$

where $l_{i,2D}$ is the cumulative length along the horizontal path from the transmitting point to the *i*-th interaction point. For paths containing only reflection and diffraction, this extension method yields a unique analytical solution for any order; for paths containing refraction or diffuse scattering, the same principle can be used to obtain approximate propagation paths. The effectiveness of this extension method is validated indirectly through the measurement-based comparison in Section 5: the measurement scenarios are real urban streets in which the transmitting antenna is deployed at two different heights (roadside ground level and overpass top), and if the vertical-dimension extension were ineffective, the simulation-to-measurement agreement of 5.6 to 8.5 dB reported in Section 5 could not be obtained. An independent full-three-dimensional validation remains as future work. It should be noted that this method inherits the cylindrical symmetry assumption: when vertical-plane multipath components (such as ground-wall coupled reflections) contribute significantly to the total received power, the 2.5D approximation introduces systematic bias. A quantitative discussion of this limitation boundary is provided in Section 6.

### 3.8. Electric Field Computation for a Single Propagation Path

**Figure 9:**
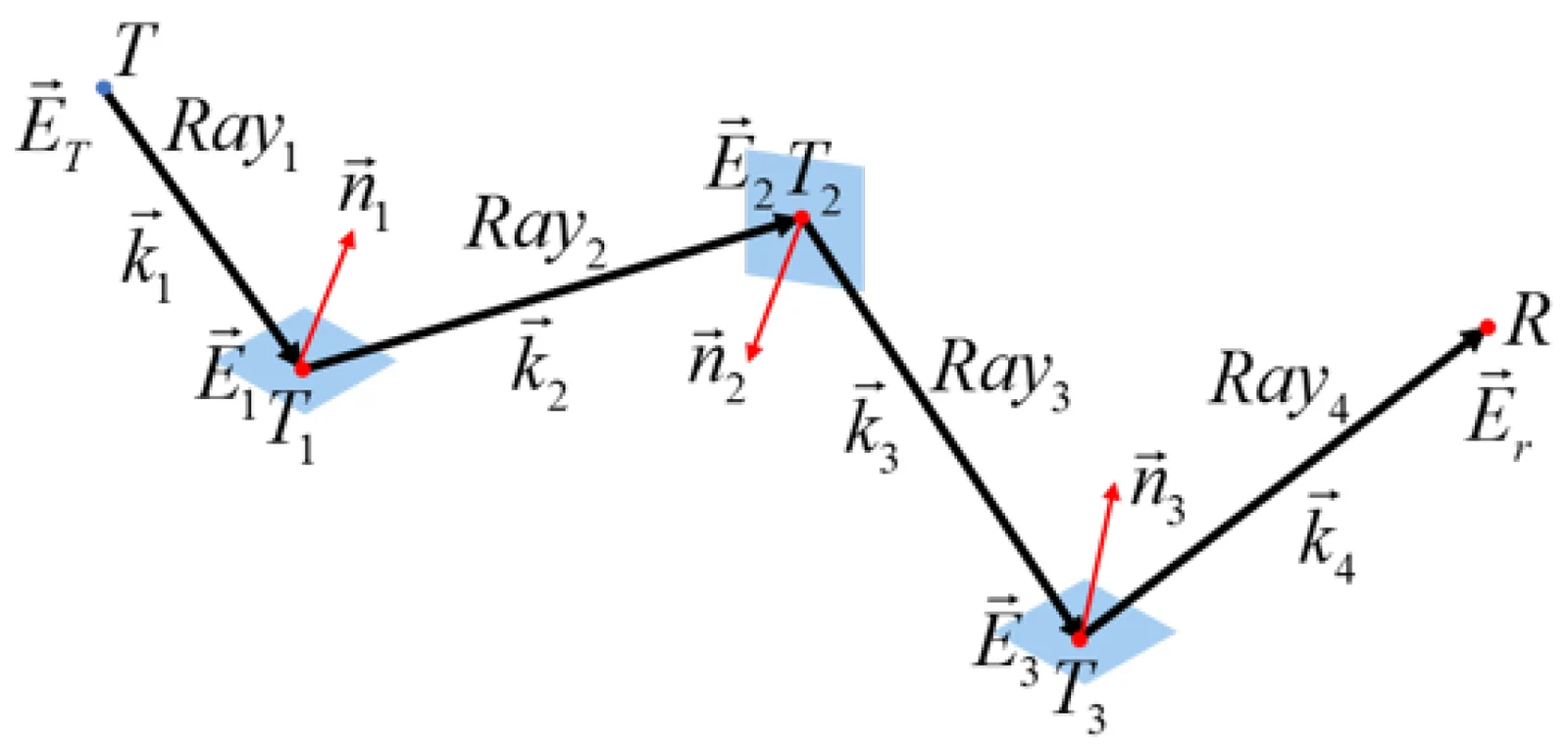
Illustration of ray tracing for a single propagation path. (The black segments denote the ray segments, the red arrows the unit normals of the interacting faces, the light blue patches the faces hit by the rays, and the blue dots the transmitter and the receiver.).

Backward path reconstruction determines the complete propagation sequence of each path and the geometric coordinates of each interaction point. After the spatial length of each segment is obtained through quasi-three-dimensional extension (see Section 3.7), the electric field contribution of the path to the receiving point can be computed. As shown in Figure 9, a typical propagation path originates from the transmitter, undergoes several propagation interactions such as reflection, refraction, diffraction, or diffuse scattering, and ultimately arrives at the receiver. It consists of *m* end-to-end line segments, each corresponding to a ray $Ray_{i}$ with length $r_{i}$; $T_{i}$ denotes the intersection point of $Ray_{i}$ with the *i*-th interacting face or corner; ${\vec{n}}_{i}$ is the normal vector of the *i*-th face. The wave vector of the *i*-th segment is ${\vec{k}}_{i}=k_{c,i}{\hat{v}}_{i}$, where ${\hat{v}}_{i}$ is the unit vector in the propagation direction and $k_{c,i}=\omega \sqrt{\mu_{c,i}\varepsilon_{c,i}}$ is the propagation constant in the medium traversed by the path segment.

Let ${\vec{E}}_{i}$ denote the electric field vector at the start of the *i*-th segment. The electric field at each interaction point is computed according to the following recurrence relation:

(8) $${\vec{E}}_{i}=As_{i}\Gamma_{i-1}{\vec{E}}_{i-1}e^{-j{\vec{k}}_{i}r_{i}},\;i=1,2,\ldots ,m$$

where $\Gamma_{i-1}$ is the propagation coefficient matrix determined by the propagation mechanism (reflection, refraction, diffraction, or diffuse scattering) at the (i−1)-th interaction point, and $As_{i}$ is the spreading factor: for the first segment, $As_{1}=1/r_{1}$, and for subsequent segments, $As_{i}=r_{i-1}/\left(r_{i-1}+r_{i}\right)$, characterizing the amplitude attenuation caused by spherical wave spreading. The initial electric field ${\vec{E}}_{1}$ is determined from the antenna radiation field according to the transmitting pattern.

Regarding the vectorial characteristics of wave propagation, it should be emphasized that the two-dimensional treatment in HFB-RTPM is confined to the path search stage. Before the electric field is computed, every path is first converted into a complete three-dimensional path through the quasi-three-dimensional extension described in Section 3.7, after which the polarization is evaluated according to the actual three-dimensional geometry. Specifically, the reflection and refraction coefficient matrices in $\Gamma_{i-1}$ are computed from the Fresnel equations, resolving the field into the components parallel and perpendicular to the plane of incidence; the diffraction coefficient follows the uniform theory of diffraction with the three-dimensional incidence and diffraction angles at the edge; and the diffuse scattering amplitude is assigned according to the actual scattering direction. The total field is therefore obtained as a vector sum of these polarization-resolved contributions, so that the vectorial nature of wave propagation is fully preserved, and the approximation of a single transverse-electric or transverse-magnetic eigen-polarization inherent to a purely two-dimensional field computation is avoided.

The single-path electric field vector at the receiving point is obtained from the last recurrence step as ${\vec{E}}_{R}=As_{m}\Gamma_{m-1}{\vec{E}}_{m-1}e^{-j{\vec{k}}_{m}r_{m}}$. After correction by the receiving antenna pattern, the received electric field ${\vec{E}}_{r,i}$ of this path is obtained, and its instantaneous received power is $P_{r,i}={\left|{\vec{E}}_{r,i}\right|}^{2}/\left(2Z_{0}\right)$, where $Z_{0}$ is the free-space wave impedance. The above components together constitute the complete HFB-RTPM propagation prediction framework.

## 4. Simulation Validation

To comprehensively validate the effectiveness of HFB-RTPM and evaluate the modeling quality of each propagation mechanism individually, this section designs four groups of progressive simulation experiments covering all propagation mechanisms: reflection comparison verifies the accuracy and efficiency advantages of the hybrid framework, refraction comparison verifies the multi-mechanism coupling management capability, diffraction comparison verifies the completeness of deterministic corner modeling, and mmWave diffuse scattering comparison verifies the physical validity of the dual-lobe scattering strategy. All simulations in this section were implemented in C++ and executed on a single-thread basis on a platform equipped with a 13th-generation Intel Core i7-13700H CPU (Intel Corporation, Santa Clara, CA, USA) and 64 GB of RAM; all reported computation times refer to this platform. Each subsection is organized according to the structure of experimental setup, quantitative results, and propagation physics analysis.

### 4.1. Accuracy and Efficiency Validation of Reflection Propagation

**Figure 10:**
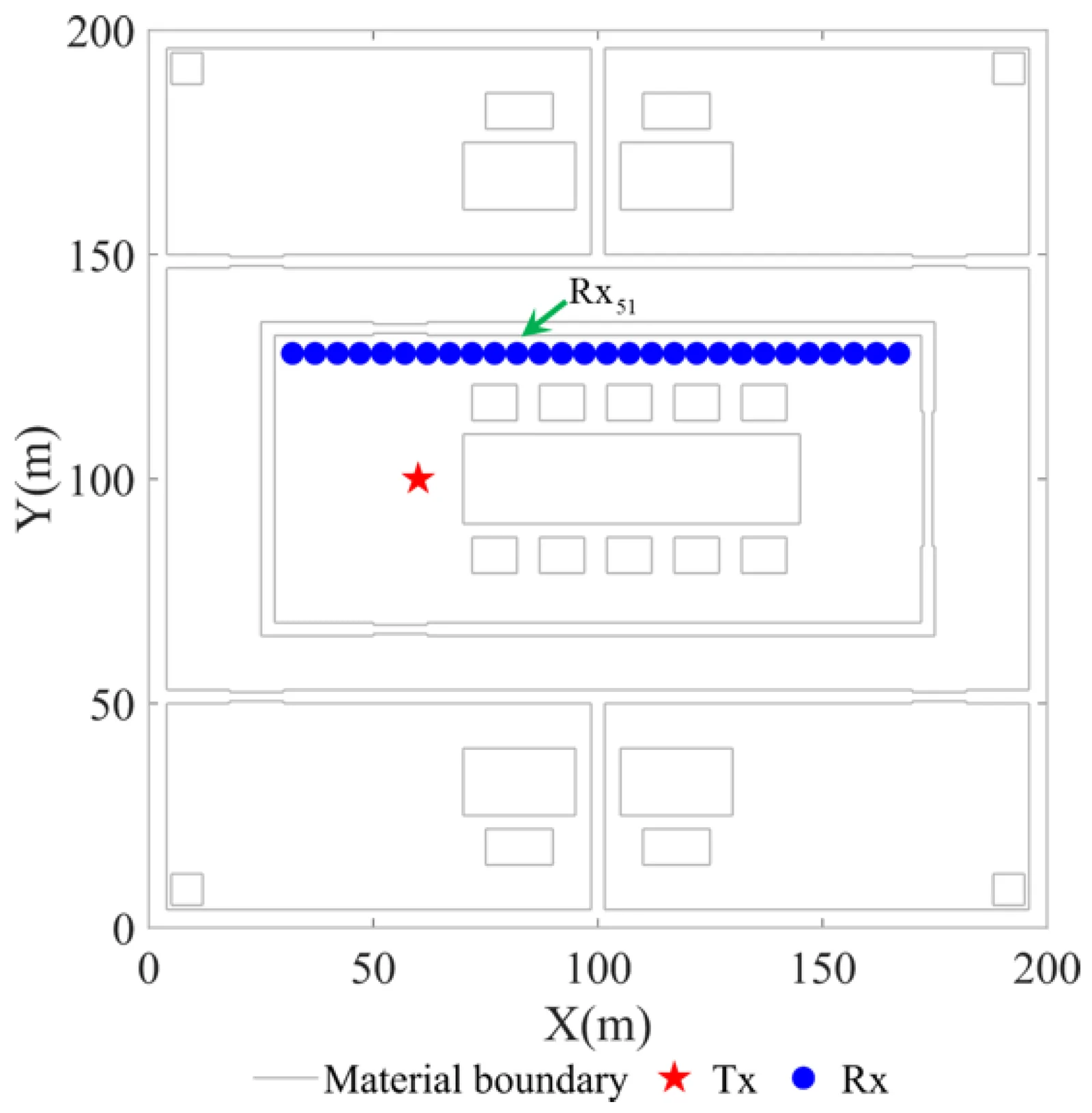
Schematic diagram of transmitter and receiver antenna positions.

This experiment adopts a typical two-dimensional indoor scenario of 200 m × 200 m (the scenario geometric modeling result is shown in Figure 1), containing 180 geometric elements and 5 ITU material types (concrete, wood, plywood, marble, metal), with a 5 GHz dipole antenna as the transmitter and 1 mW transmit power. The parameters are set as $N_{RA}=8$, $N_{RC}=0$, $N_{RD}=0$, $N_{RE}=0$, and simulation is performed under the pure reflection mechanism only. Two classical two-dimensional channel prediction methods are selected as benchmarks: the image-method-based 2D prediction model (IM-2D) provides the theoretically exact solution for reflection propagation and serves as the accuracy reference; the shooting-and-bouncing-ray-based 2D prediction model (SBR-2D), configured with 3 million rays and a 0.1 mm reception sphere radius, represents the most commonly used deterministic method in engineering practice. The transmitting antenna is located at coordinates (60.0 m, 100.0 m), and the receiving antennas are distributed along a horizontal route from (32.0 m, 128.0 m) to (168.0 m, 128.0 m) at 1.0 m intervals, totaling 137 receivers. The positions of the transmitting and receiving antennas are shown in Figure 10.

**Figure 11:**
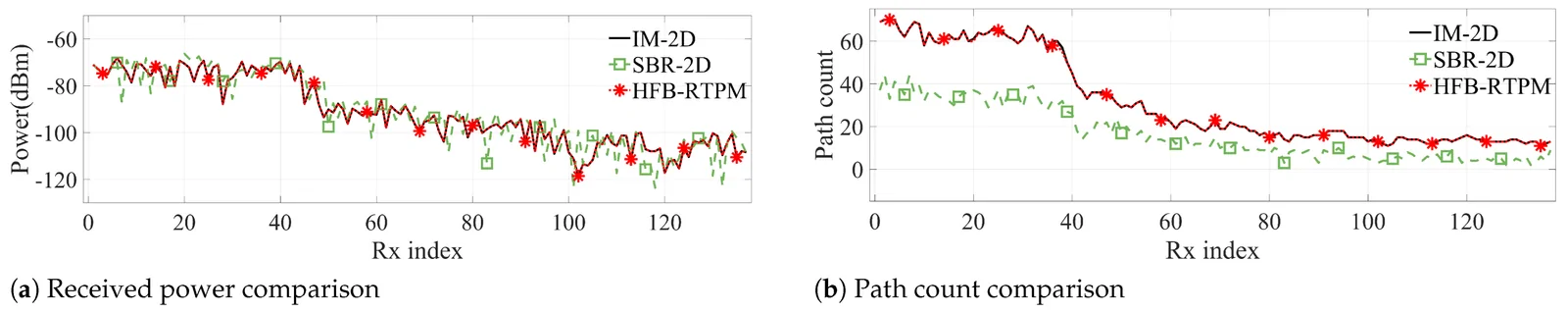
Reflection propagation comparison at $N_{RB}=7$.

**Link-level comparison:** Link-level simulations are conducted under the conditions of maximum numbers of reflections $N_{RB}$ set to 5, 6, and 7, and the results are shown in Figure 11. HFB-RTPM captures 99.73%, 99.77%, and 99.81% of the paths found by IM-2D at $N_{RB}=5$, 6, and 7, respectively, and the root mean square error (RMSE) of the received power (with IM-2D results as the reference) remains stable at 0.05 dB, which is negligible. In contrast, the path completeness rate of SBR-2D continuously decreases from 63.96% to 52.47% as the reflection order increases, and the RMSE consistently exceeds 6.4 dB. Its path omission originates from the accumulation and amplification of reception sphere approximation errors at high-order reflections, as well as the difficulty of discrete ray sampling in covering all effective propagation directions. In terms of computation time, HFB-RTPM requires only 0.28–0.45 s, which is lower than 0.60–5.91 s for IM-2D and 20.01–28.84 s for SBR-2D.

**Figure 12:**
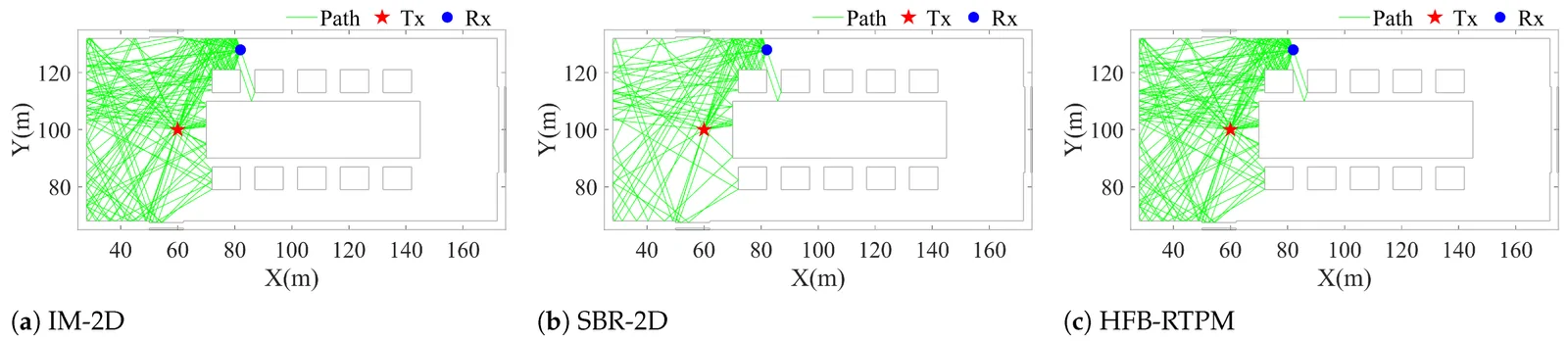
Multipath propagation paths for ${\mathrm{Rx}}_{51}$ at $N_{RB}=7$.

**Figure 13:**
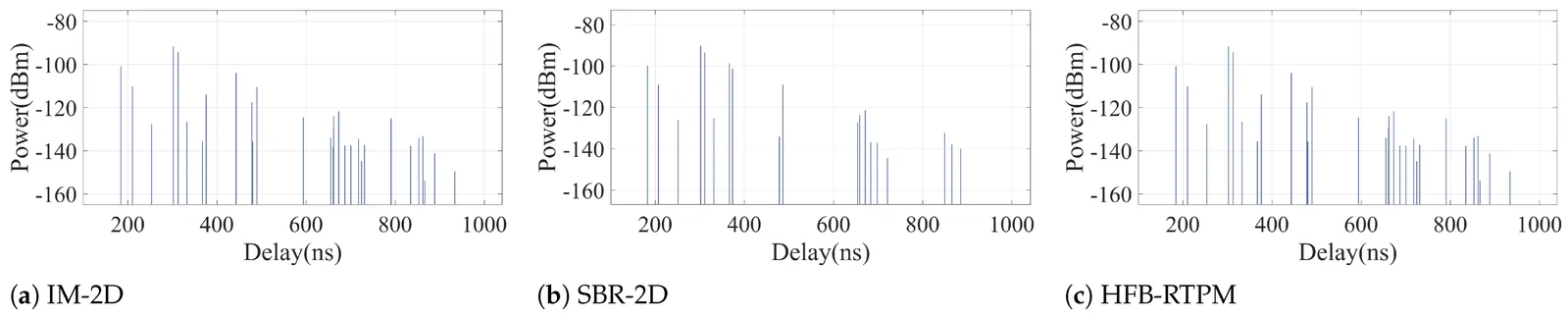
Impulse response for ${\mathrm{Rx}}_{51}$ at $N_{RB}=7$.

Figure 12 and Figure 13 further present the multipath propagation paths and power delay profile comparison for ${\mathrm{Rx}}_{51}$ under the condition $N_{RB}=7$. Figure 12 shows that the ray path geometry, reflection order, and number of paths of HFB-RTPM are completely consistent with those of IM-2D, whereas SBR-2D misses multiple high-order reflection paths. The impulse response in Figure 13 further shows that SBR-2D not only captures fewer paths but also exhibits noticeable deviations in the power of some multipath components.

**Figure 14:**
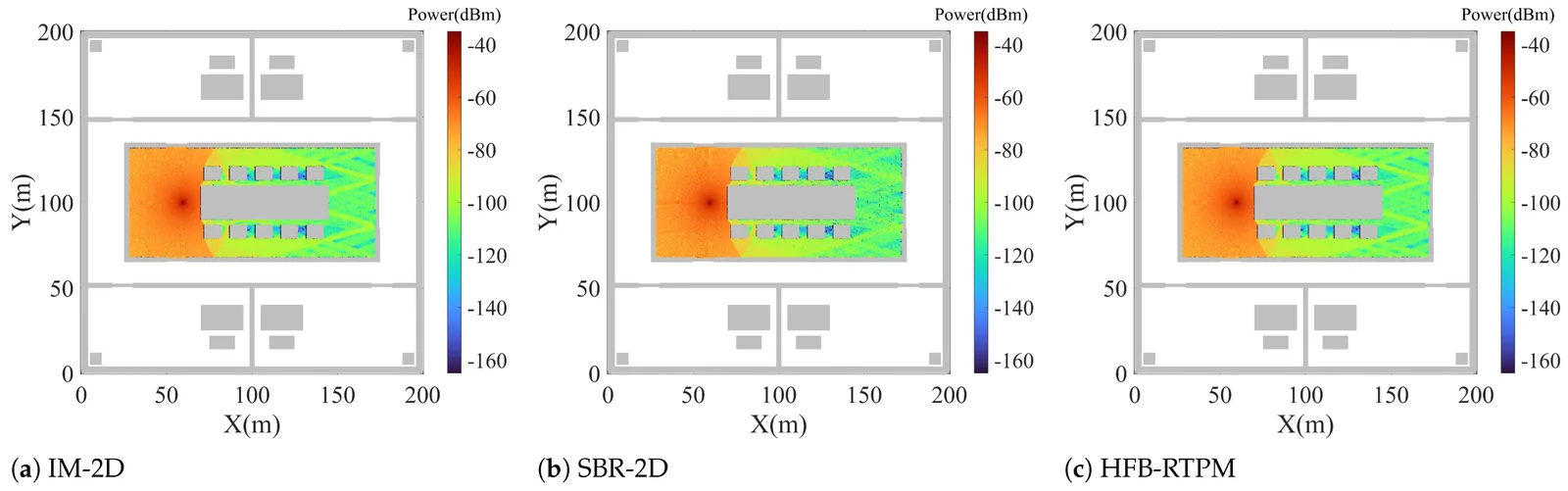
Coverage prediction at $N_{RB}=7$.

**Large-scale coverage prediction:** Coverage prediction is performed within a rectangular coverage area containing 148,289 receivers. The distribution of power prediction deviations between HFB-RTPM and IM-2D is as follows: 86.86% of the receiver points have deviations within 1 dB, only 0.09% in the 1–7 dB range, and 13.05% have deviations exceeding 7 dB (under the condition $N_{RB}=7$). The coverage prediction heatmaps are shown in Figure 14: spatially, the color transition patterns (representing the spatial variation of received power) of HFB-RTPM and IM-2D are almost completely consistent, both accurately characterizing the shadow regions formed by wall occlusion and the bright zones filled by reflected paths. In contrast, SBR-2D exhibits numerous local voids with significantly lower power in the far-field region, reflecting the path omission problem of its discrete ray sampling under high-order reflections.

The quantitative comparisons at the link and coverage levels described above are summarized in Table 2 and Table 3. Approximately 13% of the receiver points in HFB-RTPM have deviations exceeding 7 dB; these points are mainly concentrated in the regions farthest from the transmitting antenna, where the effective propagation paths are all extreme cases of seventh-order reflections. Each path accounts for a large proportion of the total power, and a small number of omissions can lead to noticeable power deviations. Considering that IM-2D already requires 21.95 ms per point in this scenario, HFB-RTPM achieves 86.86% 1 dB accuracy coverage and 99.8% path completeness with a per-point time of only 0.12 ms, and the total execution time is only approximately 0.5% of that of IM-2D, which already meets practical engineering requirements. This advantage originates from the parallelizable nature of the forward ray tube tree construction and the on-demand filtering strategy of the backward reconstruction.

Taken together, the link-level and coverage-level simulation results show that HFB-RTPM achieves prediction accuracy nearly equivalent to that of IM-2D under pure reflection conditions at an extremely low path omission cost, while improving computational efficiency by approximately two orders of magnitude, thereby validating the effectiveness of the forward–backward hybrid framework in reflection propagation modeling.

### 4.2. Validation of Refraction and Reflection-Refraction Hybrid Propagation

**Figure 15:**
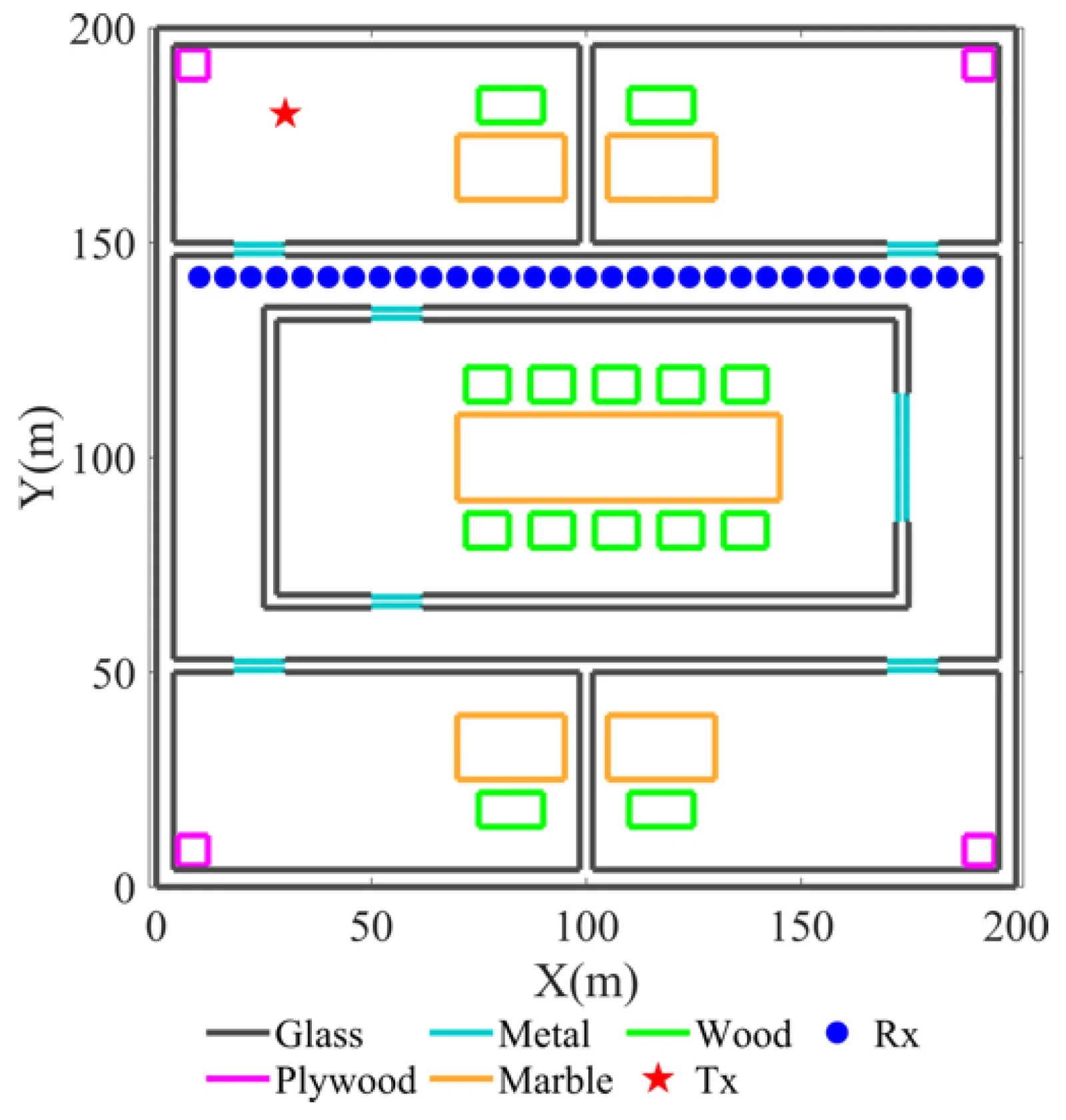
Environmental material information and antenna position diagram for refraction propagation validation.

To focus on the validation of the refraction mechanism, the concrete material in the scenario is replaced by glass to enhance the penetration effect, and the frequency is set to 1.5 GHz, while other simulation parameters remain unchanged (the scenario and antenna positions are shown in Figure 15). Since IM-2D only supports reflection, the benchmark method for this experiment is SBR-2D. It should be emphasized that SBR-2D is used here only as an engineering reference for path completeness and computational efficiency, not as an accuracy benchmark: because of the reception-sphere approximation inherent to SBR-2D, its own prediction error is non-negligible. For the refraction mechanism, since IM-2D is inapplicable and no analytical or measurement-derived reference is directly available, the validation in this subsection relies on the path completeness and the physical continuity of the multi-layer air-glass-air penetration (the continuous attenuation gradient across the layered media) as indirect evidence, and the reader is referred to the measurement-based comparison in Section 5, which involves realistic wall penetration, for a complementary assessment. A wave-method or dedicated measurement validation of the refraction mechanism is identified as future work.

**Figure 16:**
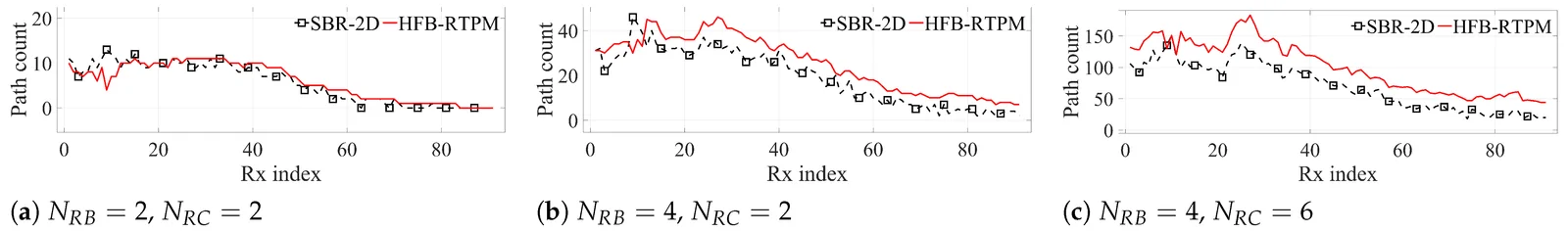
Path count comparison under different $N_{RB}$ and $N_{RC}$.

Simulations are performed under various $N_{RB}/N_{RC}$ parameter combinations, and the path count comparison results are shown in Figure 16. The three subfigures correspond to increasing complexity from low-order ($N_{RB}=2$, $N_{RC}=2$) to high-order ($N_{RB}=4$, $N_{RC}=6$) conditions, while a pure refraction baseline ($N_{RB}=0$, $N_{RC}=6$) is provided in Table 4. Under low-order conditions, the difference in path count between HFB-RTPM and SBR-2D is relatively small. As the complexity increases, the advantage of HFB-RTPM gradually becomes apparent: at $N_{RB}=4$ and $N_{RC}=6$, it generates 9168 paths, 42.7% more effective paths than the 6425 paths of SBR-2D. The detailed quantitative comparison is summarized in Table 4.

**Figure 17:**
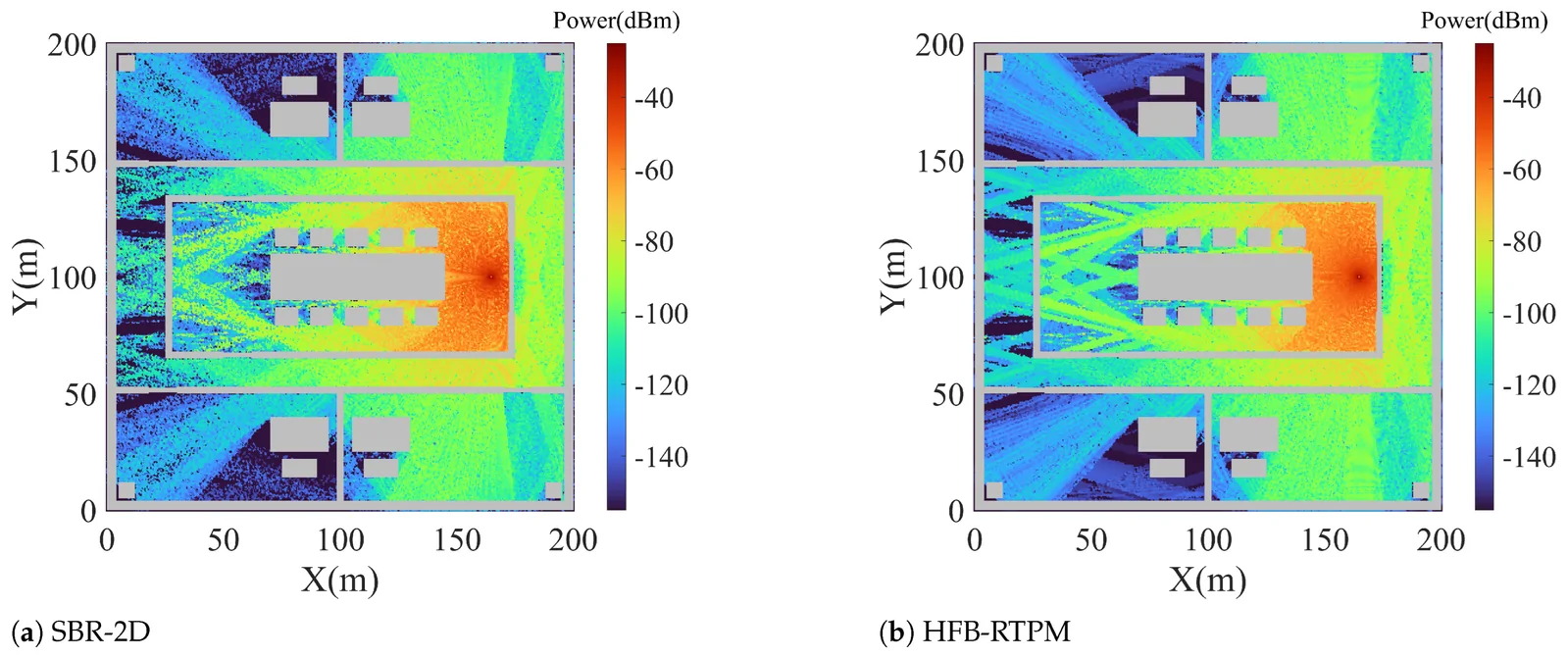
Coverage prediction comparison for refraction hybrid propagation ($N_{RB}=4$, $N_{RC}=6$).

The coverage prediction heatmaps including refraction paths are shown in Figure 17, and the quantitative statistics are summarized in Table 5. In the large-scale coverage prediction involving 160,801 receivers, the proportion of effectively covered receivers for HFB-RTPM is slightly higher than that for SBR-2D under all conditions. The difference in computational efficiency is more pronounced: under the condition $N_{RC}=6$, the total time for HFB-RTPM is 516.33 s with an average of 3.21 ms per point, whereas SBR-2D takes 10,924.94 s with an average of 67.94 ms per point, representing a more than 21-fold difference in efficiency.

In terms of field strength distribution, SBR-2D exhibits noticeable local voids and discontinuities in many regions, reflecting the difficulty of stably exciting high-order refraction paths through discrete ray launching. In contrast, HFB-RTPM demonstrates continuous and smooth physical attenuation gradients in the same regions, systematically generating, via the deterministic ray tube splitting mechanism, multi-order refraction and reflection-refraction hybrid paths, such as paths passing through air, glass, and air.

### 4.3. Validation of Edge Diffraction and Reflection-Diffraction Coupled Propagation

**Figure 18:**
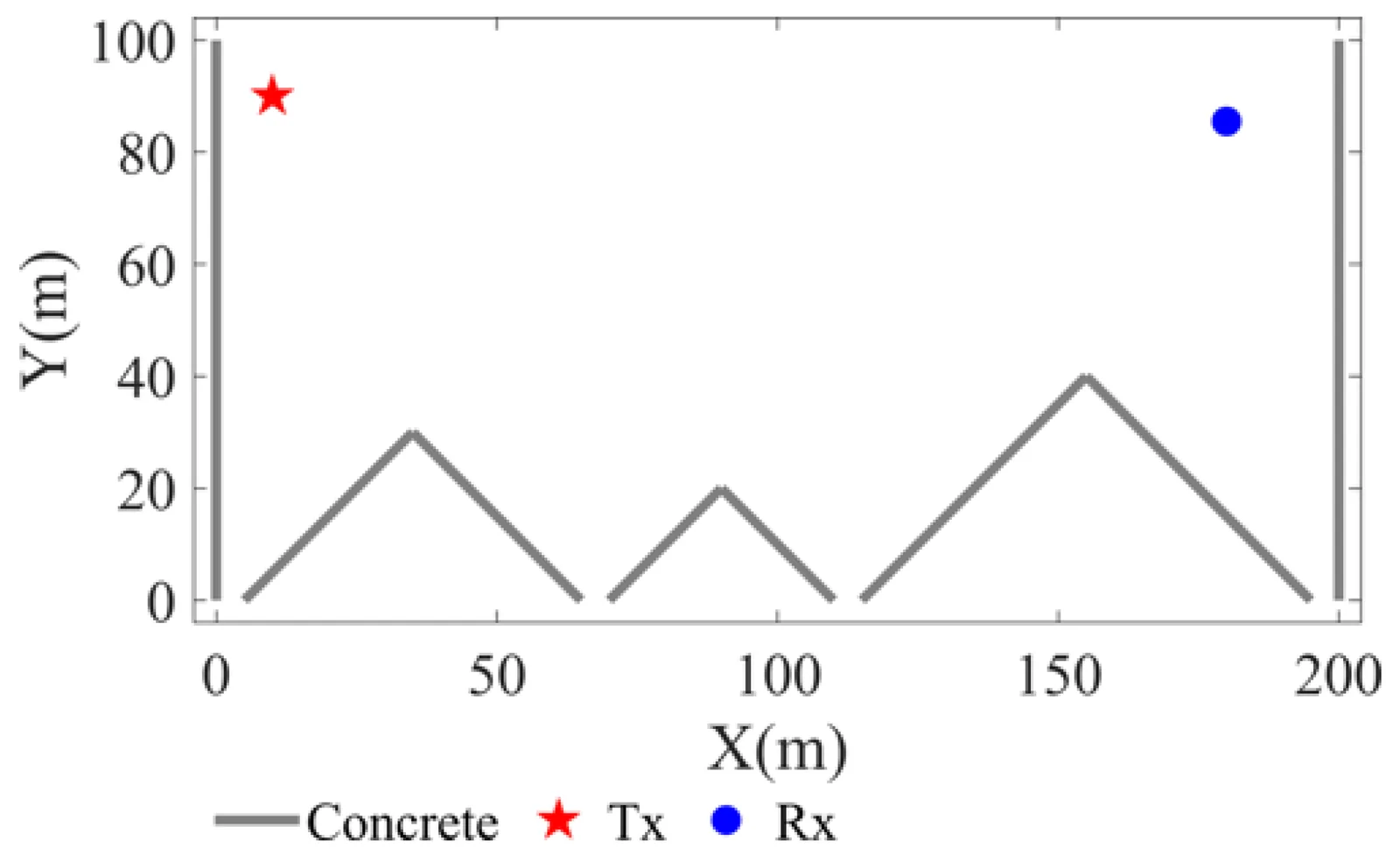
Environmental information and antenna position diagram for diffraction propagation validation.

**Small-scenario exact validation:** A simplified environment containing only 13 points, 8 line segments, and 3 corners is constructed (the scenario and antenna positions are shown in Figure 18) to verify the accuracy of diffraction modeling through per-path comparison with the analytical solution. This analytical solution is the closed-form result of the uniform theory of diffraction, that is, an analytical special solution of the wave method, and therefore constitutes an accuracy benchmark that is more rigorous than the image method and independent of the discrete-launching approximation of SBR-2D.

**Figure 19:**
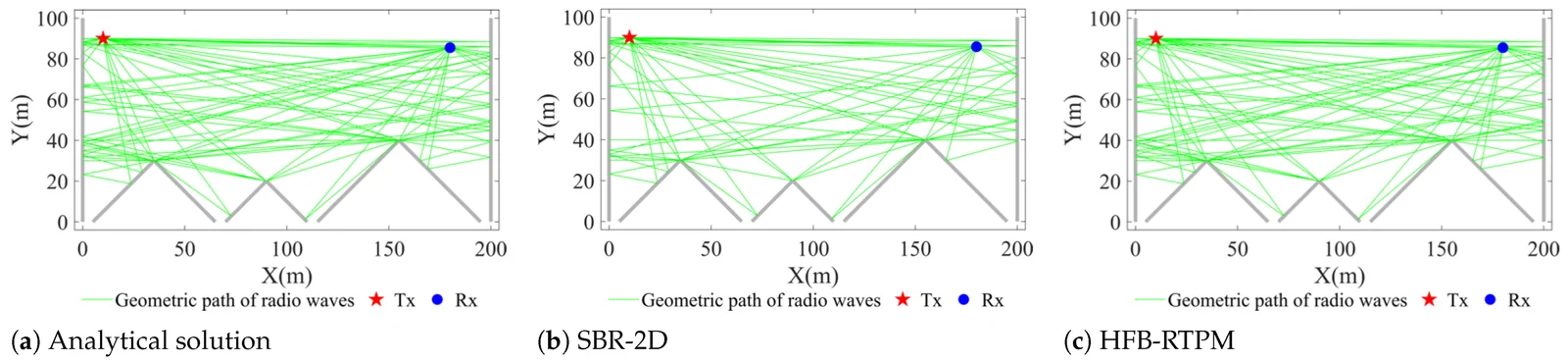
Diffraction path validation at $N_{RB}=2$, $N_{RD}=2$.

Under a total of 8 parameter combinations with $N_{RB}=0∼3$ and $N_{RD}=1∼2$, HFB-RTPM achieves 100% path completeness (verified against the analytical solution) under all conditions, whereas SBR-2D can achieve 100% under low-order conditions but drops sharply to 60.47% as complexity increases (e.g., $N_{RB}=3$, $N_{RD}=1$). Figure 19 presents a typical multipath comparison using $N_{RB}=2$, $N_{RD}=2$ as an example: the propagation paths of the analytical solution and HFB-RTPM are completely consistent in terms of geometric configuration, interaction point positions, and path count, whereas SBR-2D misses 41 paths that are predominantly high-order reflection-diffraction coupled paths (completeness rate only 73.72%). The detailed comparison of the 8 parameter combinations is summarized in Table 6.

**Figure 20:**
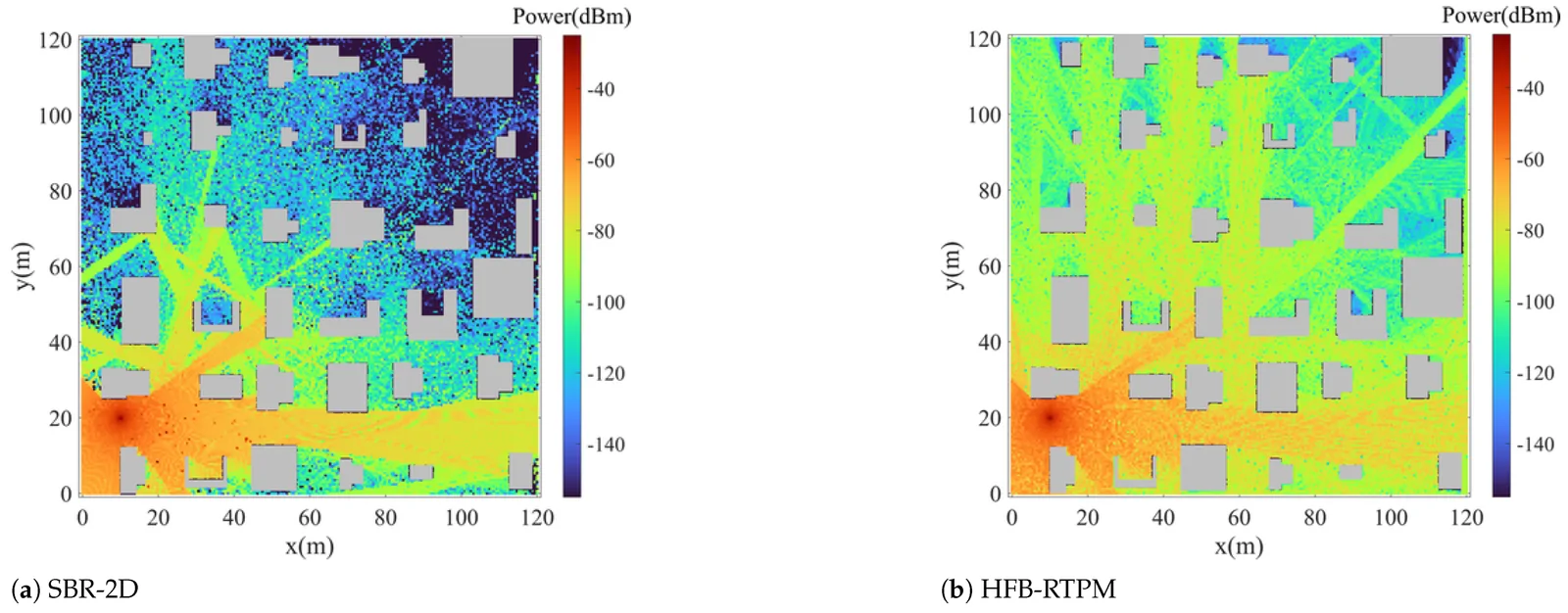
Coverage prediction for diffraction at $N_{RB}=2$, $N_{RD}=2$.

**Large-scale urban coverage prediction:** For a 120 m × 120 m dense urban block with 40,401 receivers at 3 GHz, the coverage prediction heatmap comparison is shown in Figure 20 and the quantitative statistics are summarized in Table 7. Under the commonly used engineering configuration of $N_{RD}=1$, the effective coverage proportion (received power greater than −140 dBm) of HFB-RTPM reaches 74.63%, whereas that of SBR-2D is only 45.51%, with an approximately 20-fold difference in diffraction-related computational efficiency (5.69 s vs. 114.22 s). Even when the diffraction order is elevated to 2, HFB-RTPM still maintains a significant advantage: coverage proportion 78.45% vs. 61.72%, with an approximately 3.6-fold efficiency gap.

The expanded coverage area exhibited by SBR-2D in some regions is not a manifestation of path completeness but rather a non-physical gain introduced by the reception sphere approximation, which may lead to power overestimation. HFB-RTPM, based on strict geometric intersection determination, avoids such approximation errors. The above small-scenario exact validation and large-scale urban coverage prediction results demonstrate that the corner diffraction modeling of HFB-RTPM can achieve a 100% path completeness rate without sacrificing computational efficiency, significantly outperforming SBR-2D, which relies on discrete ray sampling.

### 4.4. Robustness Validation of mmWave Diffuse Scattering

To validate the effectiveness of the diffuse scattering mechanism, the prediction results of HFB-RTPM at two mmWave frequency bands, 28 GHz and 73 GHz, are compared with the NYU statistical urban microcell model derived from extensive measurements in Manhattan [21]. This model constitutes a measurement-derived statistical benchmark rather than a ray-method reference of the same origin, so the comparison below provides an accuracy reference that is independent of the ray-tracing family of methods. The NYU model adopts the close-in free-space reference distance path loss model with log-normal shadow fading:

(9) $$PL^{\mathrm{CI}}\left(f,d\right)=FSPL\left(f,d_{0}\right)+10n\log_{10}\;\left(d/d_{0}\right)+X_{\sigma },\;d\geq d_{0}$$

where $FSPL\left(f,d_{0}\right)=20\log_{10}\left(4\pi d_{0}/\lambda \right)$ is the free-space path loss at the reference distance $d_{0}=30$ m, *n* is the path loss exponent, and $X_{\sigma }∼N\left(0,\sigma^{2}\right)$ is a zero-mean Gaussian random variable capturing large-scale shadow fading. The values of *n* and σ for each frequency band and LoS/NLoS condition are listed in Table 8. In the following comparison, only the deterministic mean path loss (i.e., $PL^{\mathrm{CI}}$ without the $X_{\sigma }$ term) is used as the reference, since the objective is to evaluate the accuracy of the deterministic prediction against the underlying propagation trend, rather than to replicate the random shadowing realizations present in any individual measurement.

**Figure 21:**
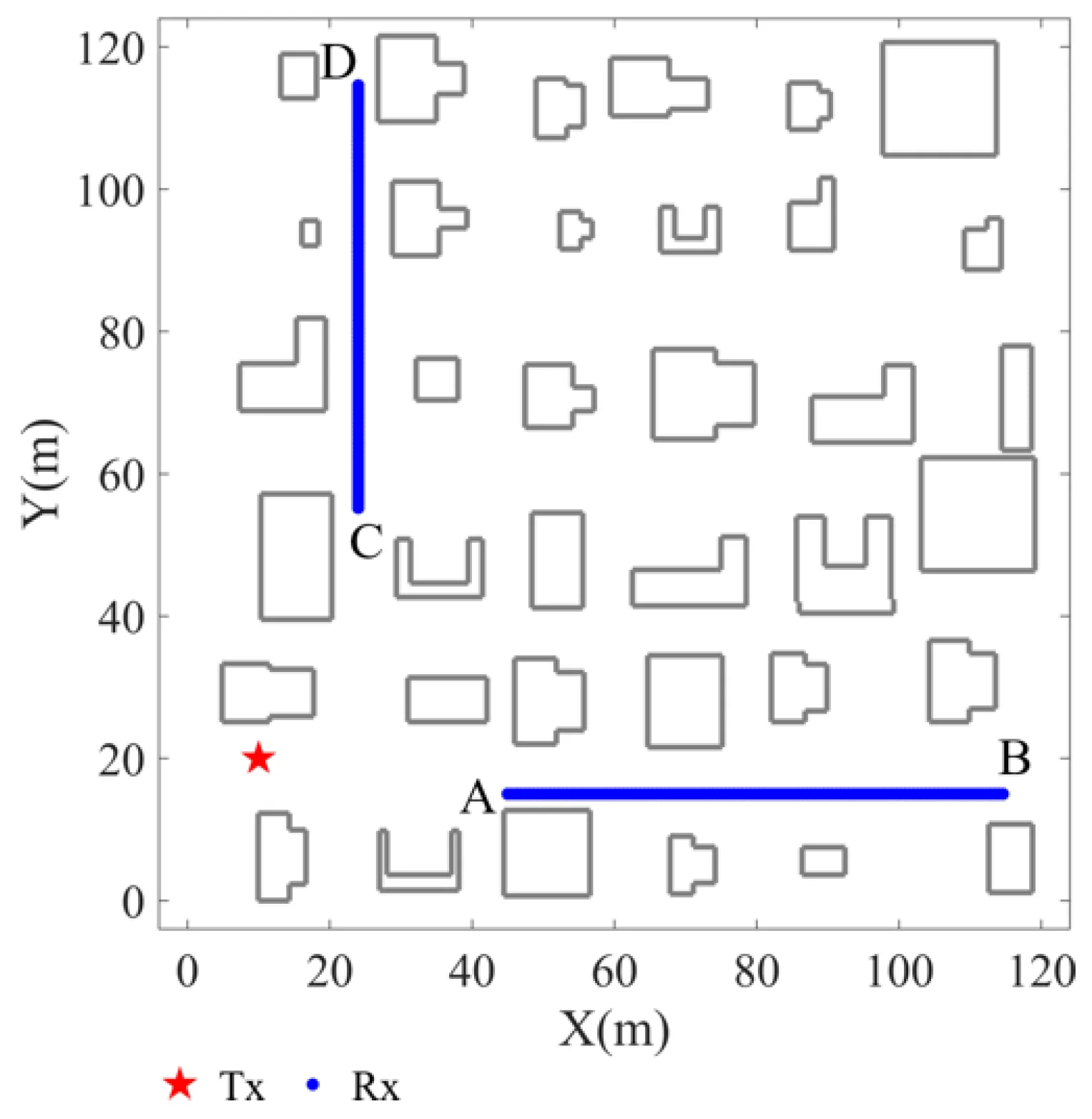
Urban micro-scenario and antenna position diagram for diffuse scattering validation.

The urban micro-scenario and antenna positions are shown in Figure 21, where the transmitting antenna is placed at (10.0 m, 20.0 m), the line-of-sight (LoS) route AB (A at (45.0 m, 15.0 m), B at (115.0 m, 15.0 m)), and the NLoS route CD (C at (24.0 m, 55.0 m), D at (24.0 m, 115.0 m)). The HFB-RTPM parameters are configured as $N_{RA}=4$, $N_{RB}=3$, $N_{RC}=0$, $N_{RD}=1$.

**Figure 22:**
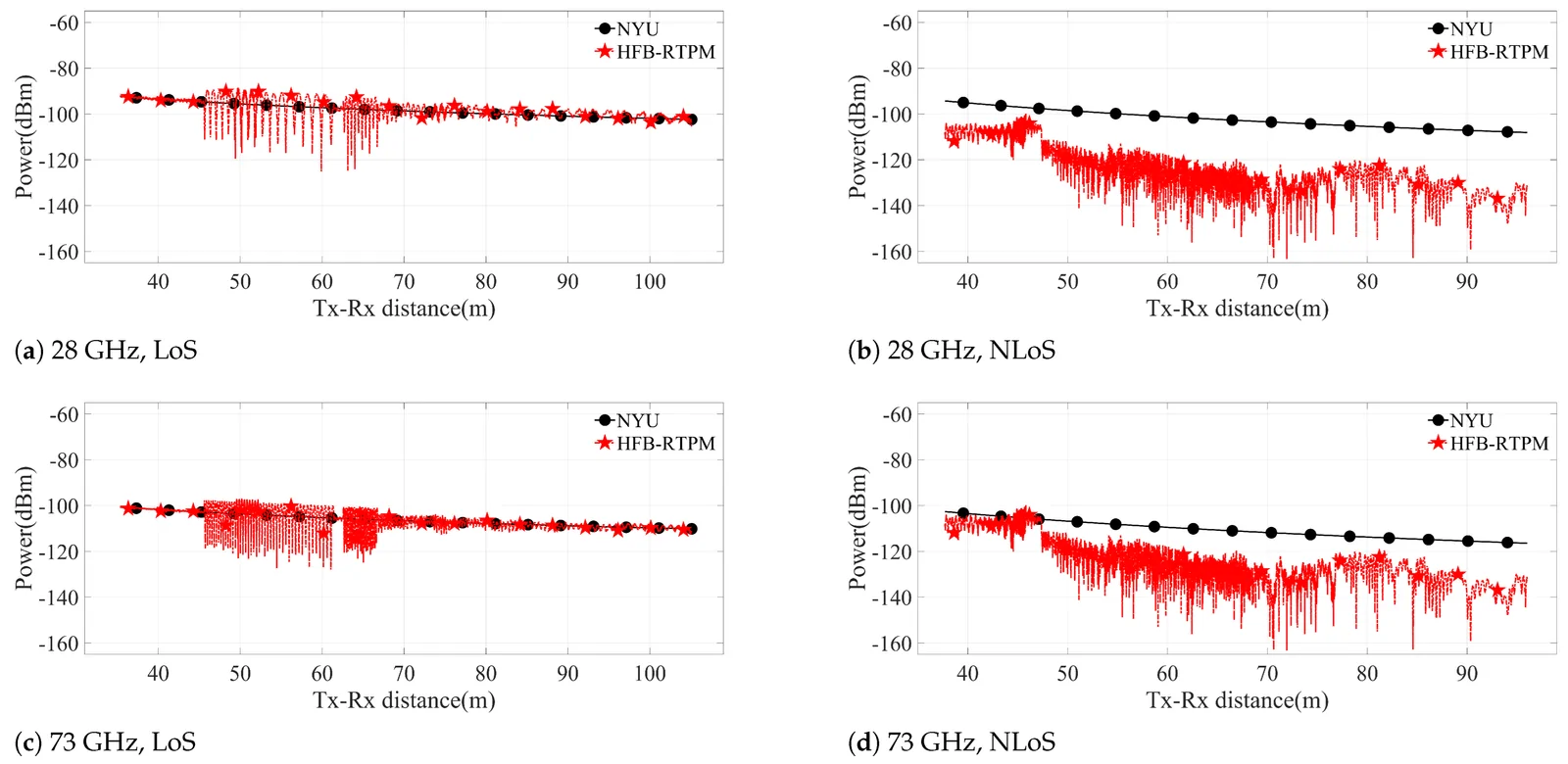
Received power without diffuse scattering.

**Prediction without diffuse scattering:** Figure 22 presents the power curve comparison between HFB-RTPM (pure deterministic, without diffuse scattering enabled) and the NYU mean path loss model, where the solid lines represent the predicted received power of HFB-RTPM at each receiver point and the dashed lines represent the NYU reference curves computed from Equation (9) without the shadow fading term $X_{\sigma }$. On the LoS route, HFB-RTPM achieves RMSE values of 3.43 dB and 3.22 dB at the two frequency bands, showing good agreement with the NYU mean path loss. On the NLoS route, however, the RMSE values reach 23.86 dB and 16.19 dB, respectively, indicating that the purely deterministic model substantially underestimates the received power in NLoS conditions. Figure 22b,d show that the predicted received power on the NLoS route is far below the NYU reference, confirming that without diffuse scattering, the deterministic framework cannot account for the scattered energy contributed by rough building surfaces, which constitutes a significant portion of the total received power in mmWave NLoS scenarios. Notably, the NYU shadow fading standard deviation σ for NLoS reaches 9.7 dB and 7.9 dB at 28 and 73 GHz, respectively; these large values reflect the inherent variability of NLoS propagation that deterministic models without diffuse scattering cannot reproduce.

**Figure 23:**
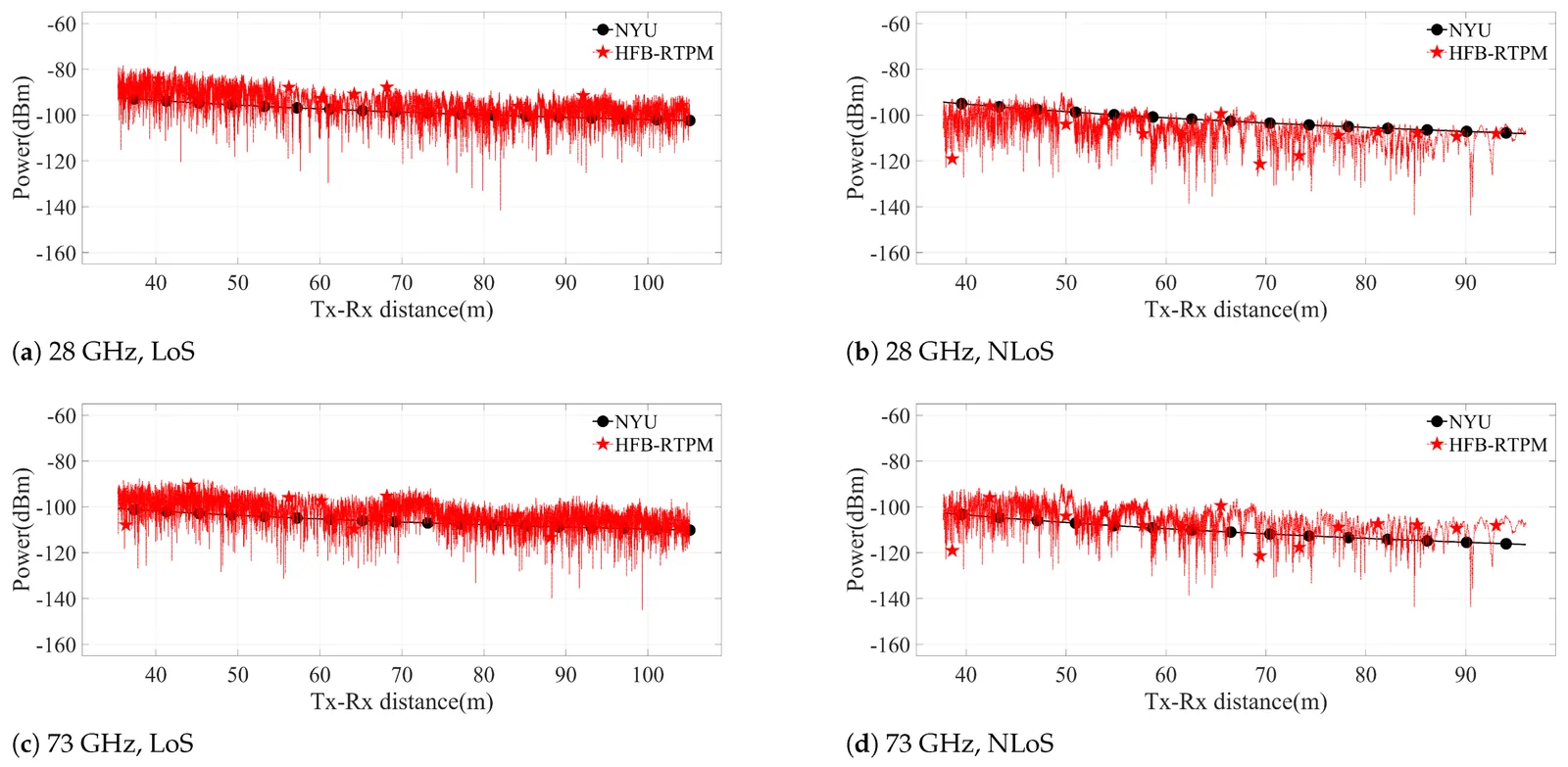
Received power with diffuse scattering.

**Prediction with diffuse scattering:** The NLoS prediction accuracy is substantially improved after incorporating the dual-lobe diffuse scattering model, as shown in Figure 23. The RMSE values decrease from 23.86 to 6.87 dB (28 GHz) and from 16.19 to 6.82 dB (73 GHz). Although the RMSE on the LoS route increases slightly (from approximately 3.3 to 6.3 dB), this modest increase is expected because the smooth-surface assumption in the pure deterministic model already provides a reasonable approximation for LoS propagation, and the additional scattered components introduce minor fluctuations around the mean trend. Crucially, the final RMSE values in NLoS conditions (6.87 dB at 28 GHz and 6.82 dB at 73 GHz) both fall within the corresponding NYU shadow fading standard deviations (σ=9.7 dB at 28 GHz and σ=7.9 dB at 73 GHz), indicating that the residual prediction error after incorporating diffuse scattering is within the natural variability of the propagation environment.

The detailed quantitative comparison is summarized in Table 9. A single model configuration is simultaneously effective at both 28 and 73 GHz without per-band recalibration, demonstrating the physical consistency and cross-band robustness of the proposed diffuse scattering model.

The above results demonstrate that HFB-RTPM, through a unified diffuse scattering parameterization framework, significantly compensates for the prediction deficiencies of the purely deterministic model in mmWave NLoS scenarios. After incorporating diffuse scattering, the NLoS RMSE values at both 28 and 73 GHz lie within the NYU shadow fading standard deviations, confirming that the residual error is consistent with the inherent physical variability of the propagation channel. Meanwhile, the cross-band consistency of the diffuse scattering parameters facilitates multi-band joint simulation without per-band recalibration.

## 5. Measurement-Based Propagation Validation

### 5.1. Measurement System and Propagation Scenarios

To validate the predictive capability of HFB-RTPM in real propagation environments, two sets of channel measurement experiments, designated as street-A and street-B, were conducted in a typical densely built-up urban block. The measurement platform employed an NI-USRP-2954 software-defined radio platform with a PXIe-MXIe chassis, and link calibration was performed at both the transmitter and receiver ends prior to the measurements.

Table 10 summarizes the key measurement parameters. The four frequency points (2.3 GHz, 3 GHz, 4 GHz, and 5.9 GHz) were selected to cover the current cellular communication and WiFi/unlicensed bands and verify the cross-band adaptability of the model. The two transmit antenna configurations (roadside ground-level deployment and overpass-top deployment) simulate typical installation patterns of urban communication infrastructure to assess the model’s adaptability to different transmitter heights. After power delay profile processing, a 1 ms time window was applied to average the instantaneous received power, suppressing small-scale fading fluctuations.

**Figure 24:**
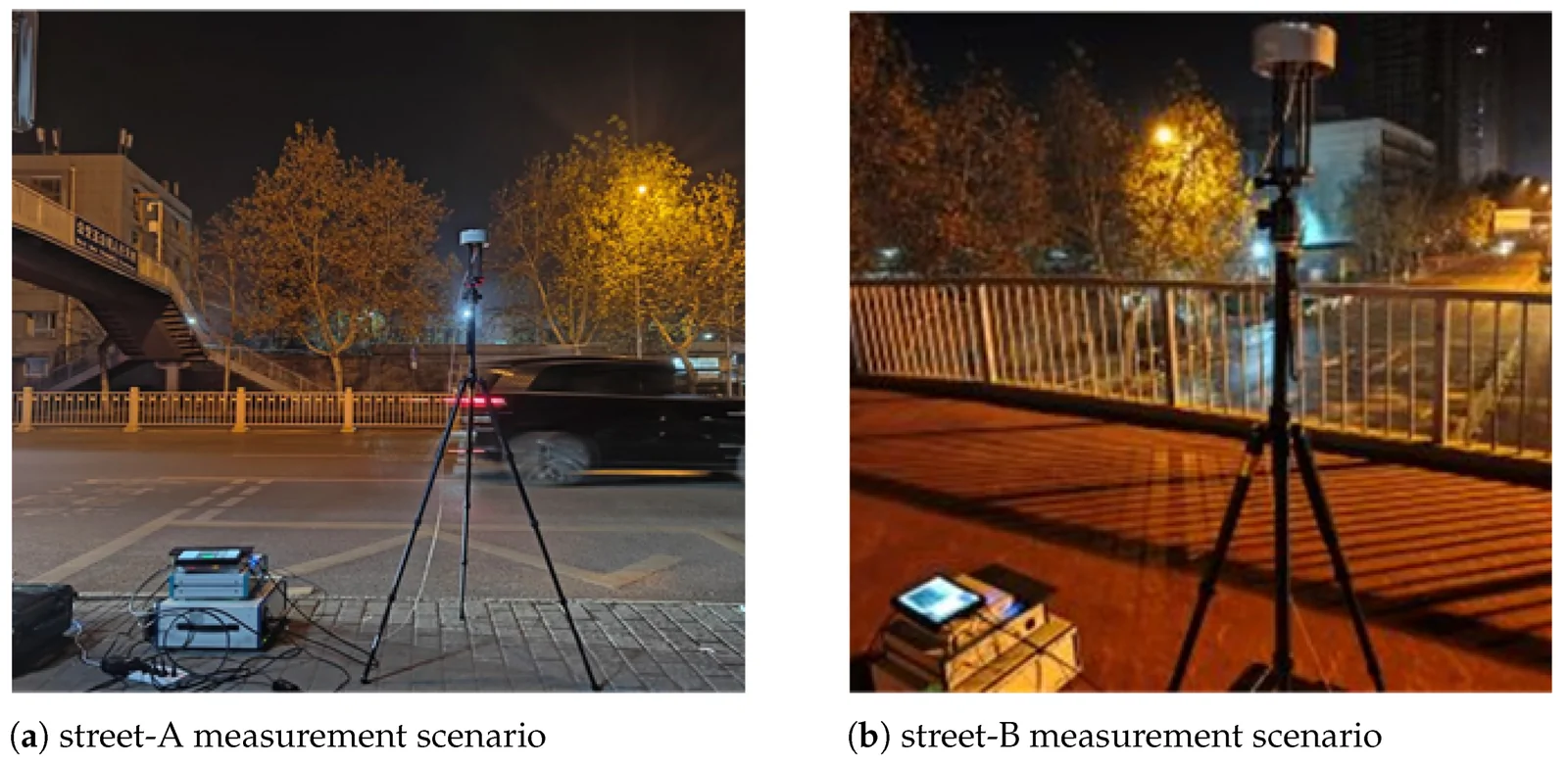
Channel measurement scenarios in the densely built-up urban block.

**Figure 25:**
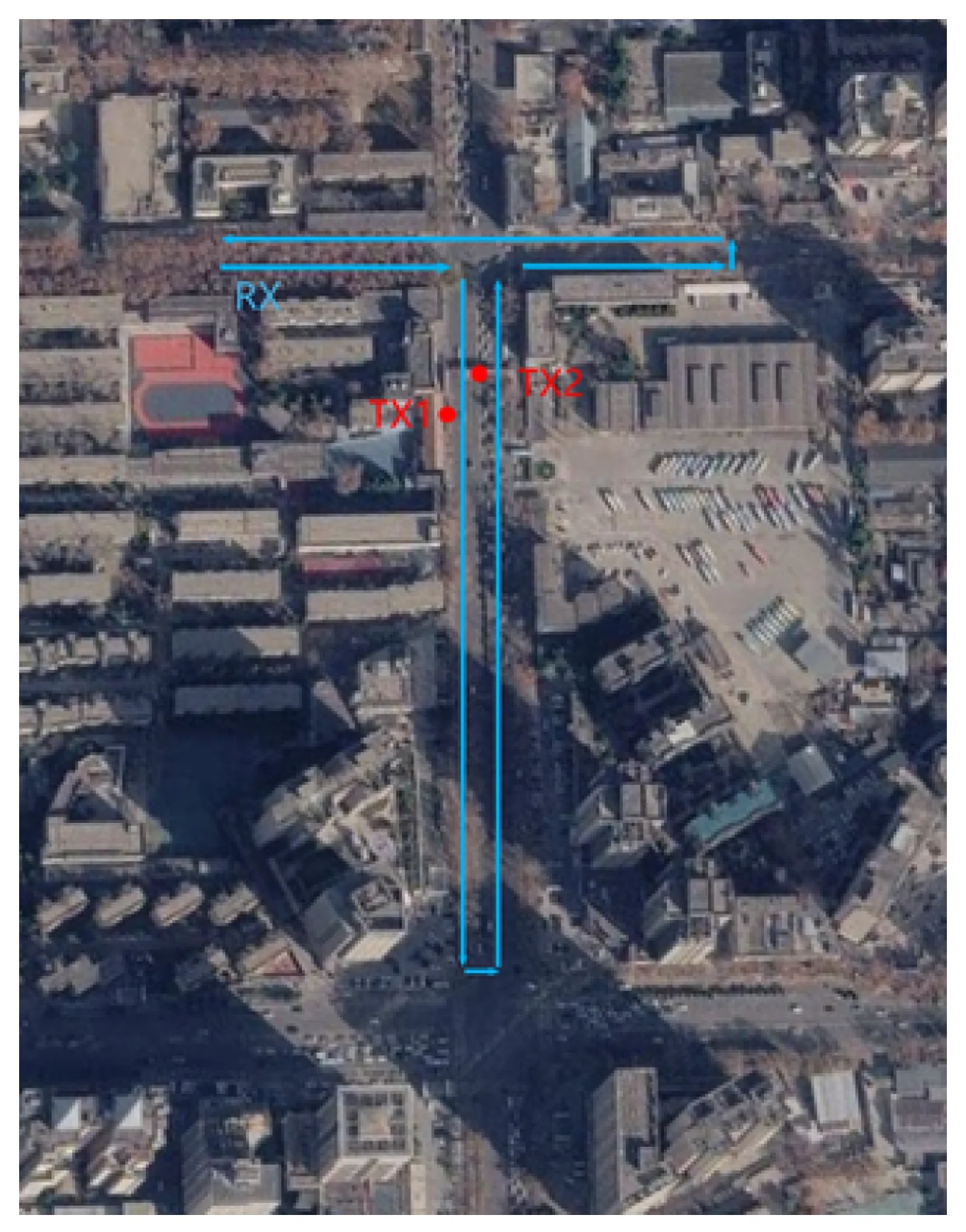
Schematic diagram of transmit antenna positions and measurement route.

The site photographs and antenna positions for the two propagation scenarios are shown in Figure 24 and Figure 25. The transmitter ${\mathrm{Tx}}_{1}$ was deployed beside the road to simulate a typical roadside unit installation, while the transmitter ${\mathrm{Tx}}_{2}$ was deployed on the overpass to simulate an elevated roadside infrastructure deployment. The receiving vehicle traveled along the direction indicated by the red arrow in the figure, along which the transceiver repeatedly underwent transitions between LoS and NLoS conditions.

### 5.2. Simulation Versus Measurement Comparison

**Figure 26:**
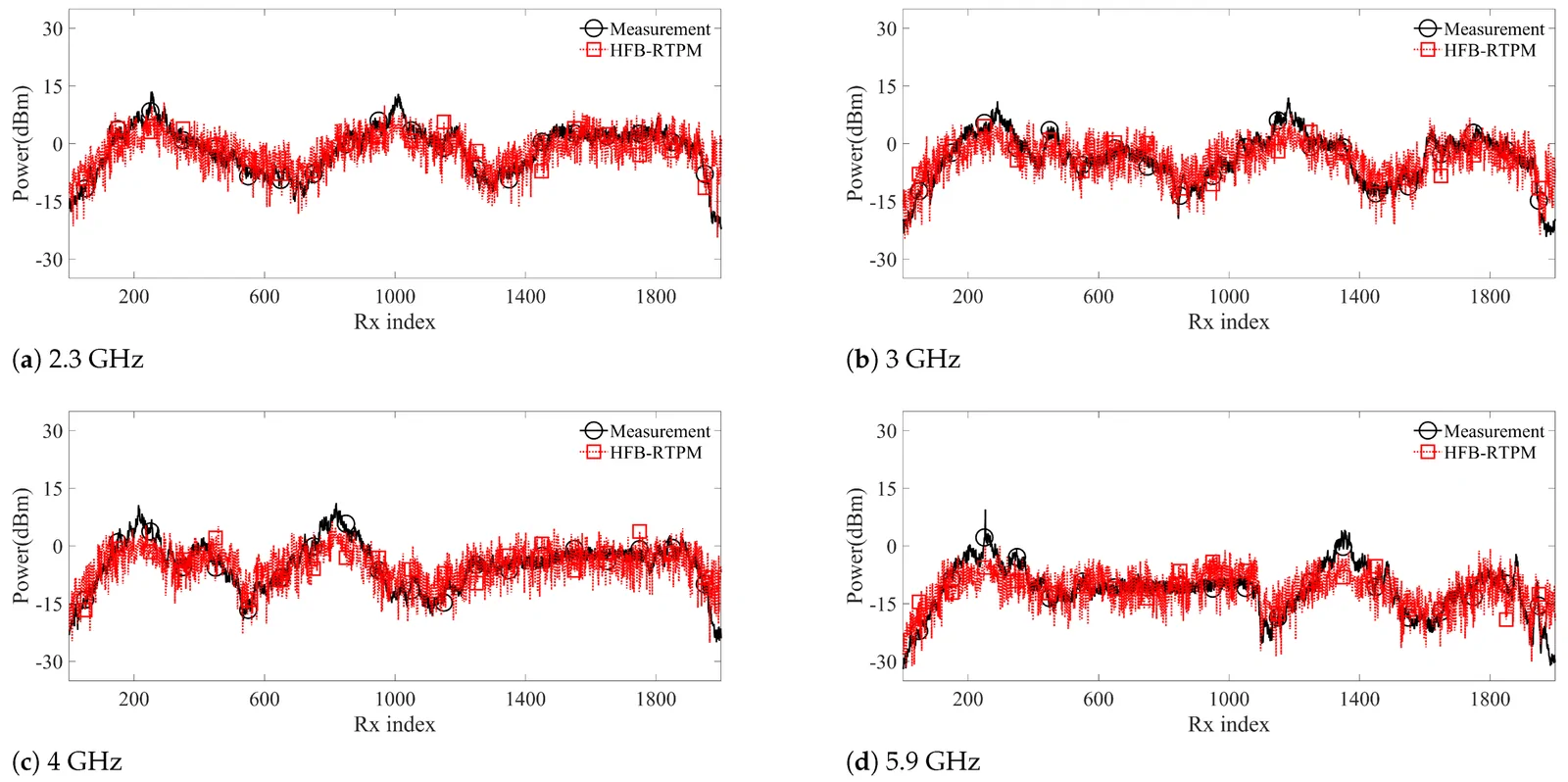
Comparison of HFB-RTPM simulated and measured power curves in street-A.

**Figure 27:**
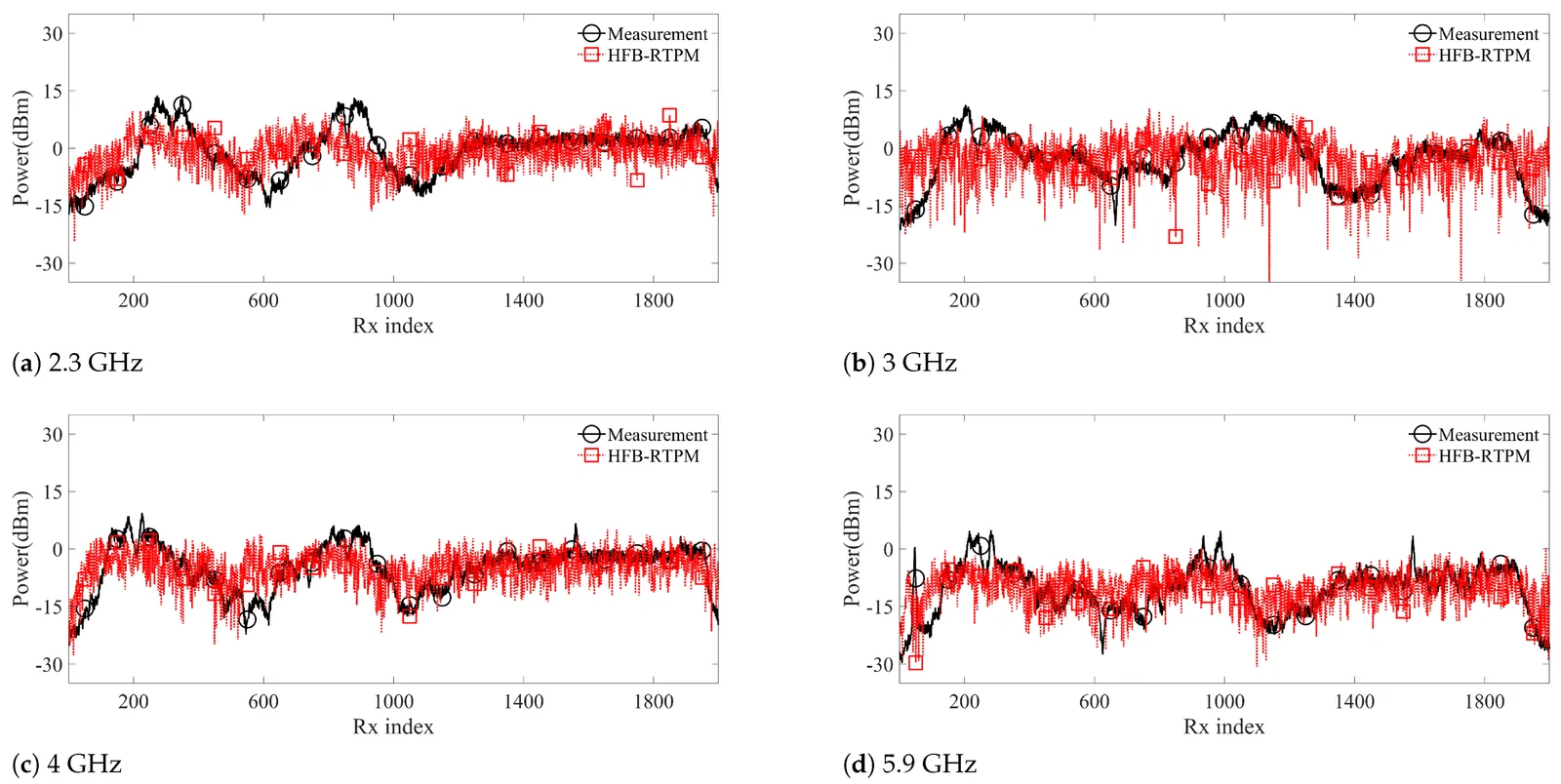
Comparison of HFB-RTPM simulated and measured power curves in street-B.

The HFB-RTPM simulation results for the street-A and street-B scenarios are compared against the measured data, with the results shown in Figure 26 and Figure 27.

Across all eight comparison groups (4 frequency points × 2 scenarios), the simulated curves exhibit good agreement with the measured curves in terms of overall trend and waveform, successfully reproducing the key feature of LoS-to-NLoS transition along the measurement route. The RMSE statistics are summarized in Table 11: in the street-A scenario, the RMSE values for the four frequency points range from 5.61 to 6.08 dB; in the street-B scenario, they range from 7.35 to 8.45 dB.

The overall RMSE of street-B is higher than that of street-A, which stems from its transmitter’s elevated placement on the overpass, resulting in a wider propagation coverage area and more complex blocking patterns. The highest RMSE in street-B occurs at 3 GHz (8.45 dB), likely due to more abundant diffraction path components at this frequency that cannot be fully characterized by the 2.5D approximation in the vertical dimension. The RMSE in street-A increases monotonically with frequency (5.61 → 6.08 dB), whereas street-B does not exhibit such a trend, reflecting the differences in the composition of propagation mechanisms under different transmitter heights. The fact that the RMSE under both transmitter heights remains within an acceptable range, and that the error pattern varies with the transmitter height in an interpretable way, provides indirect evidence for the validity of the quasi-three-dimensional extension: were the vertical-dimension extension ineffective, the simulation-to-measurement agreement reported here could not be achieved.

Four main error sources are identified. First, the 2.5D dimensionality reduction approximation is the primary error source: HFB-RTPM is centered on horizontal-plane ray tracing with equivalent approximations in the vertical dimension, while the vertical-plane reflection and diffraction components are more pronounced for the elevated transmitter in street-B, leading to larger errors. Second, systematic deviations exist between the ITU recommended values and the actual electromagnetic parameters of the building materials. Third, the simplification of actual building shapes in the scene geometric modeling introduces local geometric errors. Fourth, mobile scatterers (pedestrians, vehicles) present during the measurement were not modeled, constituting an additional source of local prediction errors.

From an engineering standpoint, the RMSE across the two experiments and four frequency points is controlled within 5.6–8.5 dB, falling within the acceptable accuracy range of typical deterministic propagation prediction models [30]. The consistent results across the four frequency bands demonstrate the effectiveness of the material frequency-dependent model based on ITU-R P.2040, and the acceptable prediction accuracy under the two transmitter heights demonstrates that the 2.5D quasi-three-dimensional approach adapts well to urban propagation scenarios.

## 6. Discussion and Conclusions

Table 12 provides a quantitative and qualitative comparison of HFB-RTPM with two representative methods across three dimensions: accuracy, efficiency, and mechanism completeness. The image method provides theoretically exact solutions in purely reflective scenarios but cannot handle diffuse scattering on rough surfaces and refraction in lossy media, limiting its application to idealized environments. The launching ray method surpasses the image method in mechanism generality, but due to its discrete launching and reception sphere approximation design, its path omission is systematic rather than random: for *N*-th order reflection paths, the angular sampling density of rays must increase as $O(N^{2})$ to maintain capture probability, causing the completeness rate of higher-order paths to decay rapidly with order (as shown in Table 6, where SBR-2D achieves only 64.5% completeness under $N_{RB}=3,N_{RD}=2$ conditions). HFB-RTPM resolves this issue at the architectural level: forward tree construction covers all possible propagation interactions, and backward tracing only filters paths that actually arrive, so completeness is unaffected by propagation order. This architectural-level improvement (rather than parameter tuning or heuristic acceleration) is the essential feature that distinguishes it from existing methods.

On the choice between 2.5D and full 3D, the 2.5D method provides a favorable trade-off between accuracy and computational efficiency in cylindrically symmetric scenarios (e.g., dense building blocks): it preserves precise geometric processing capability in the horizontal plane, avoids the computational bottleneck where the number of faces grows from O(N) to $O(N^{2})$ in full 3D ray tracing, while recovering elevation propagation information through analytical extension in the vertical dimension. However, when multipath components in the vertical plane (such as ground reflections and ceiling reflections) account for a high proportion of total received power, the 2.5D approximation introduces systematic bias, which is the physical reason that street-B’s overall RMSE is approximately 2 dB higher than that of street-A. As a practical guideline, when vertical-plane multipath contributions are expected to exceed approximately 30% of the total received power, a condition commonly encountered in scenarios with low transmitter heights, wide street canyons, or strong specular ground reflections, the full 3D HFB-RTPM-3D is recommended. Extending to the full 3D HFB-RTPM-3D requires addressing challenges such as three-dimensional ray tube splitting and face collision detection, where the 3D ray tube splitting strategy (transitioning from conical to pyramidal segmentation) is the core difficulty.

It should also be acknowledged that the ray method and the wave method both originate from the same physical field equations and should converge in the ideal limit. The mechanism-by-mechanism validation in Section 4 therefore establishes the internal consistency of the ray method in terms of path completeness and received-power accuracy, rather than a direct comparison against a full-wave solution. For scattering and diffraction, where the image method is no longer applicable, this paper adopts the analytical UTD solution (an analytical special solution of the wave method) for diffraction and the measurement-derived NYU statistical model for diffuse scattering as the accuracy references, which are already stronger than the image method and applicable to these mechanisms. The most rigorous means of verifying the accuracy in scattering and diffraction scenarios is a direct comparison against a full-wave field solution, such as a two-dimensional finite-element solution; the mesh scale and computational cost of such a full-wave solution in the present 200 m × 200 m, multi-material, high-frequency scenarios exceed the scope of this work, and this comparison is therefore identified as a separate validation study in the future work below.

The sharp drop of NLoS prediction RMSE (from 23.9/16.2 dB to 6.9/6.8 dB at 28/73 GHz) after incorporating diffuse scattering confirms that this mechanism is not an optional feature for high-frequency propagation prediction but a necessary requirement for ensuring physical plausibility. Moreover, a single model configuration proves effective at both 28 GHz and 73 GHz without per-band recalibration, suggesting that the diffuse scattering behavior is governed primarily by surface geometry rather than frequency-dependent electromagnetic properties. If this frequency independence is validated across additional bands, it will substantially reduce the parameter calibration burden in multi-band joint simulations.

From an engineering standpoint, the combination of high accuracy and high efficiency in HFB-RTPM endows it with the potential to support practical network planning and optimization: the per-point computation time for large-scale coverage prediction in reflection scenarios is as low as 0.06 ms (see Table 3), enabling tasks such as base station site optimization, channel fingerprint construction, and beam management pre-training data generation. Its accurate assessment of millimeter-wave NLoS coverage is directly relevant to base station density deployment decisions.

In summary, this paper proposes the hybrid forward–backward ray tube propagation model (HFB-RTPM), which resolves the contradiction among accuracy, completeness, and computational efficiency in deterministic multipath propagation prediction at the architectural level. Through mechanism-by-mechanism simulations and four-band measurement validation, HFB-RTPM achieves reflection and diffraction path completeness rates approaching or reaching 100%. The introduction of dual-lobe diffuse scattering reduces the mmWave NLoS prediction RMSE by 17.0 dB (28 GHz) and 9.4 dB (73 GHz), and the measured RMSE at 2.3–5.9 GHz is 5.6–8.5 dB. Regarding the verification benchmarks, the accuracy references adopted for the refraction, diffraction, and diffuse-scattering groups are, respectively, path completeness with physical continuity, the analytical UTD solution, and the measurement-derived NYU statistical model; a direct comparison against a full-wave solution, such as a two-dimensional finite-element field solution, and a dedicated measurement validation of the refraction mechanism are identified as future work. Future work may proceed in three directions: the theoretical derivation and implementation of full 3D HFB-RTPM-3D, focusing on the 3D ray tube splitting strategy and face intersection detection algorithm; the physical characterization of diffuse scattering through surface roughness statistics, replacing empirical calibration with a physically grounded parameterization; and extending the HFB-RTPM framework to emerging scenarios such as sub-terahertz frequency bands and reconfigurable intelligent surface-assisted propagation. In addition, the BVH spatial acceleration structure reduces the average complexity of a single intersection test from linear to logarithmic order, and the forward ray-tube-tree construction is inherently parallelizable; these properties give HFB-RTPM the potential to scale to larger scenarios, such as space-air-ground integrated networks (SAGIN), where a comprehensive simulation framework for propagation channel research is actively being developed [33]. Mega-constellation networks, whose robustness under geographical failure has recently been analyzed [34], likewise demand highly efficient deterministic channel prediction, which can directly benefit from the low per-point cost reported in this paper. This scalability remains to be demonstrated in future work.

## Figures and Tables

**Table 1:** Fitting coefficients of electromagnetic parameters for building materials recommended by ITU-R P.2040-3 and their electromagnetic parameters at 3 GHz.

Material	*f* (GHz)	*a*	*b*	*c*	*d*	$\varepsilon_{r}$ (3 GHz)	σ (S/m, 3 GHz)
Air	0.001–100	1	0	0	0	1.00	0
Concrete	1–100	5.24	0	0.0462	0.7822	5.24	0.109
Wood	0.001–100	1.99	0	0.0047	1.0718	1.99	0.015
Plywood	1–40	2.71	0	0.33	0	2.71	0.330
Glass	0.1–100	6.31	0	0.0036	1.3394	6.31	0.016
Marble	1–60	7.074	0	0.0055	0.9262	7.07	0.015
Metal	1–100	1	0	$10^{7}$	0	1.00	$1.0\times 10^{7}$

**Table 2:** Accuracy and efficiency comparison of HFB-RTPM with IM-2D and SBR-2D under reflection propagation conditions.

Algorithm	$N_{\mathrm{RB}}$	Total Paths	Path Completeness	RMSE (dB)	Time (s)
IM-2D	5	2583	100.00%	0	0.60
IM-2D	6	3434	100.00%	0	1.66
IM-2D	7	4309	100.00%	0	5.91
SBR-2D	5	1652	63.96%	6.78	20.01
SBR-2D	6	1981	57.69%	6.48	23.93
SBR-2D	7	2261	52.47%	6.54	28.84
HFB-RTPM	5	2576	99.73%	0.05	0.28
HFB-RTPM	6	3426	99.77%	0.05	0.34
HFB-RTPM	7	4301	99.81%	0.05	0.45

**Table 3:** Reflection propagation coverage prediction error and time comparison (148,289 receivers).

Algorithm	$N_{\mathrm{RB}}$	Error vs. IM-2D			Total Time (s)	Per-Point Time (ms)
		≤1 dB	**1–7 dB**	**>7 dB**		
IM-2D	5	/	/	/	195.07	1.32
IM-2D	6	/	/	/	1426.05	9.62
IM-2D	7	/	/	/	3255.36	21.95
SBR-2D	5	24.95%	43.26%	31.79%	820.33	5.53
SBR-2D	6	24.50%	44.07%	31.43%	903.35	6.09
SBR-2D	7	24.46%	44.33%	31.21%	986.23	6.65
HFB-RTPM	5	86.86%	0.08%	13.06%	9.02	0.06
HFB-RTPM	6	86.86%	0.09%	13.05%	15.96	0.11
HFB-RTPM	7	86.86%	0.09%	13.05%	17.15	0.12

**Table 4:** Impact of reflection and refraction orders on path count.

$N_{\mathrm{RB}}$	$N_{\mathrm{RC}}$	SBR-2D Total Paths $M_{s}$	HFB-RTPM Total Paths $M_{h}$	$M_{h}/M_{s}$
0	6	55	52	94.55%
2	2	512	545	106.45%
2	4	1213	1415	116.65%
2	6	1970	2517	127.77%
4	2	1771	2310	130.43%
4	4	5827	8291	142.29%
4	6	6425	9168	142.69%

**Table 5:** Coverage prediction results including refraction paths (160,801 receivers).

Algorithm	$N_{\mathrm{RC}}$	Rx > −140 dBm	Total Time (s)	Per-Point Time (ms)
SBR-2D	2	35.70%	2372.98	14.76
SBR-2D	4	73.04%	8629.14	53.66
SBR-2D	6	77.59%	10,924.94	67.94
HFB-RTPM	2	38.03%	35.46	0.22
HFB-RTPM	4	75.02%	349.84	2.18
HFB-RTPM	6	78.85%	516.33	3.21

**Table 6:** Impact of reflection and diffraction orders on path completeness rate.

$N_{\mathrm{RB}}$	$N_{\mathrm{RD}}$	Algorithm	Path Count	Completeness vs. Analytical
0	1	SBR-2D	4	100.00%
		HFB-RTPM	4	100.00%
0	2	SBR-2D	10	100.00%
		HFB-RTPM	10	100.00%
1	1	SBR-2D	16	84.21%
		HFB-RTPM	19	100.00%
1	2	SBR-2D	47	85.45%
		HFB-RTPM	55	100.00%
2	1	SBR-2D	34	70.83%
		HFB-RTPM	48	100.00%
2	2	SBR-2D	115	73.72%
		HFB-RTPM	156	100.00%
3	1	SBR-2D	52	60.47%
		HFB-RTPM	86	100.00%
3	2	SBR-2D	198	64.50%
		HFB-RTPM	307	100.00%

**Table 7:** Coverage prediction results including diffraction paths.

Algorithm	$N_{\mathrm{RD}}$	Rx > −140 dBm	Total Time (s)	Per-Point Time (ms)
SBR-2D	0	23.37%	6.02	0.15
SBR-2D	1	45.51%	114.22	2.83
SBR-2D	2	61.72%	4166.22	103.12
HFB-RTPM	0	23.31%	0.50	0.01
HFB-RTPM	1	74.63%	5.69	0.14
HFB-RTPM	2	78.45%	1159.94	28.71

**Table 8:** NYU urban micro-environment statistical model path loss parameters ($d_{0}=30$ m).

LoS/NLoS	Frequency (GHz)	Path Loss Exponent *n*	σ (dB)
LoS	28	2.1	3.6
LoS	73	2.0	4.8
NLoS	28	3.4	9.7
NLoS	73	3.4	7.9

**Table 9:** RMSE comparison of HFB-RTPM with the NYU model with and without diffuse scattering.

Diffuse Scattering Included	Frequency (GHz)	LoS/NLoS	RMSE (dB)
No	28	LoS	3.43
		NLoS	23.86
	73	LoS	3.22
		NLoS	16.19
Yes	28	LoS	6.21
		NLoS	6.87
	73	LoS	6.41
		NLoS	6.82

**Table 10:** Channel measurement parameter configuration in the densely built-up urban block.

Parameter	Configuration
Bandwidth (MHz)	160
Sampling rate (MHz)	200
Transmit power (dBm)	20
Positioning system	GPS + RTK (Real-Time Kinematic)
Measurement platform	NI-USRP-2954 + PXIe-MXIe chassis
Measurement frequency (GHz)	2.3, 3, 4, 5.9

**Table 11:** HFB-RTPM simulation-to-measurement RMSE for street-A and street-B (dB).

Frequency (GHz)	Street-A RMSE	Street-B RMSE
2.3	5.61	7.39
3	5.77	8.45
4	5.96	7.48
5.9	6.08	7.35

**Table 12:** Comprehensive performance comparison of HFB-RTPM with other methods.

Performance Dimension	Image Method	Launching Ray Method	HFB-RTPM
Reflection path completeness	100%	52–64%	99.8%
Diffraction path completeness	—	60–100%	100%
Refraction path capability	Not supported	Limited	Complete
Diffuse scattering support	Not supported	Limited	Unified framework
Large-scale coverage efficiency	Slow	Slow	Fast

## Data Availability

The data presented in this study are available on request from the corresponding author.
